# Sci-Seq of Human Fetal Salivary Tissue Introduces Human Transcriptional Paradigms and a Novel Cell Population

**DOI:** 10.3389/fdmed.2022.887057

**Published:** 2022-09-16

**Authors:** Devon Duron Ehnes, Ammar Alghadeer, Sesha Hanson-Drury, Yan Ting Zhao, Gwen Tilmes, Julie Mathieu, Hannele Ruohola-Baker

**Affiliations:** 1 Department of Biochemistry, School of Medicine, University of Washington, Seattle, WA, United States,; 2 Institute for Stem Cells and Regenerative Medicine, School of Medicine, University of Washington, Seattle, WA, United States,; 3 Department of Biomedical Dental Sciences, College of Dentistry, Imam Abdulrahman bin Faisal University, Dammam, Saudi Arabia,; 4 Department of Oral Health Sciences, School of Dentistry, University of Washington, Seattle, WA, United States,; 5 Department of Comparative Medicine, University of Washington, Seattle, WA, United States,; 6 Department of Bioengineering, University of Washington, Seattle, WA, United States

**Keywords:** salivary gland, human fetal development, stem cells, single-cell sequencing, exocrine gland

## Abstract

Multiple pathologies and non-pathological factors can disrupt the function of the non-regenerative human salivary gland including cancer and cancer therapeutics, autoimmune diseases, infections, pharmaceutical side effects, and traumatic injury. Despite the wide range of pathologies, no therapeutic or regenerative approaches exist to address salivary gland loss, likely due to significant gaps in our understanding of salivary gland development. Moreover, identifying the tissue of origin when diagnosing salivary carcinomas requires an understanding of human fetal development. Using computational tools, we identify developmental branchpoints, a novel stem cell-like population, and key signaling pathways in the human developing salivary glands by analyzing our human fetal single-cell sequencing data. Trajectory and transcriptional analysis suggest that the earliest progenitors yield excretory duct and myoepithelial cells and a transitional population that will yield later ductal cell types. Importantly, this single-cell analysis revealed a previously undescribed population of stem cell-like cells that are derived from SD and expresses high levels of genes associated with stem cell-like function. We have observed these rare cells, not in a single niche location but dispersed within the developing duct at later developmental stages. Our studies introduce new human-specific developmental paradigms for the salivary gland and lay the groundwork for the development of translational human therapeutics.

## INTRODUCTION

Exocrine glands, including mammary, prostate, sweat, lacrimal, and salivary, are epithelial tissues comprised of duct systems through which they secrete various factors onto adjacent surfaces. Epithelial carcinomas account for the majority of cancer cases (90%). However, identifying the tissue origin of the carcinoma is hampered by the fact that while the cancer cells are reverting to a fetal stage, the human fetal development of many exocrine glands is still poorly understood.

General exocrine gland development shares a complex stepwise process including epithelial ingrowth to form a bud, branching morphogenesis and ductal elongation, and further, secretory cell differentiation (1). It has been well established that epithelial-mesenchymal interactions (2–6), integrin- (7, 8) and fibroblast growth factor 10 (FGF10)-signaling are critical for exocrine gland morphogenesis and development (9–14). However, the mechanism of action of signaling pathways and specific transcription factors that drive cell fate decisions during different exocrine gland development have remained largely elusive. Increased accessibility to single-cell sequencing technologies has the potential to remedy this.

The salivary gland is an exocrine gland that produces and secretes saliva. In humans, there are three major types of glands, namely the parotid, the submandibular, and the sublingual glands, which produce ~90% of all saliva, and several minor salivary glands dispersed throughout the oral submucosa make up the other 10%. Saliva holds important roles in tissue repair, oral lubrication, tooth mineralization and protection, and taste (15, 16), and is a vehicle for severe acute respiratory syndrome-coronavirus 2 (SARS-CoV2) spread (17–19). Several factors can perturb the proper function of salivary glands, including Sjögren’s syndrome (20–22) and cancer and resultant radiation therapy (23). Compounding this issue is that salivary glands have been shown to have poor regenerative capacity following injury despite reports of the existence of stem-like cells in adult tissues (24).

**FIGURE 1 |:**
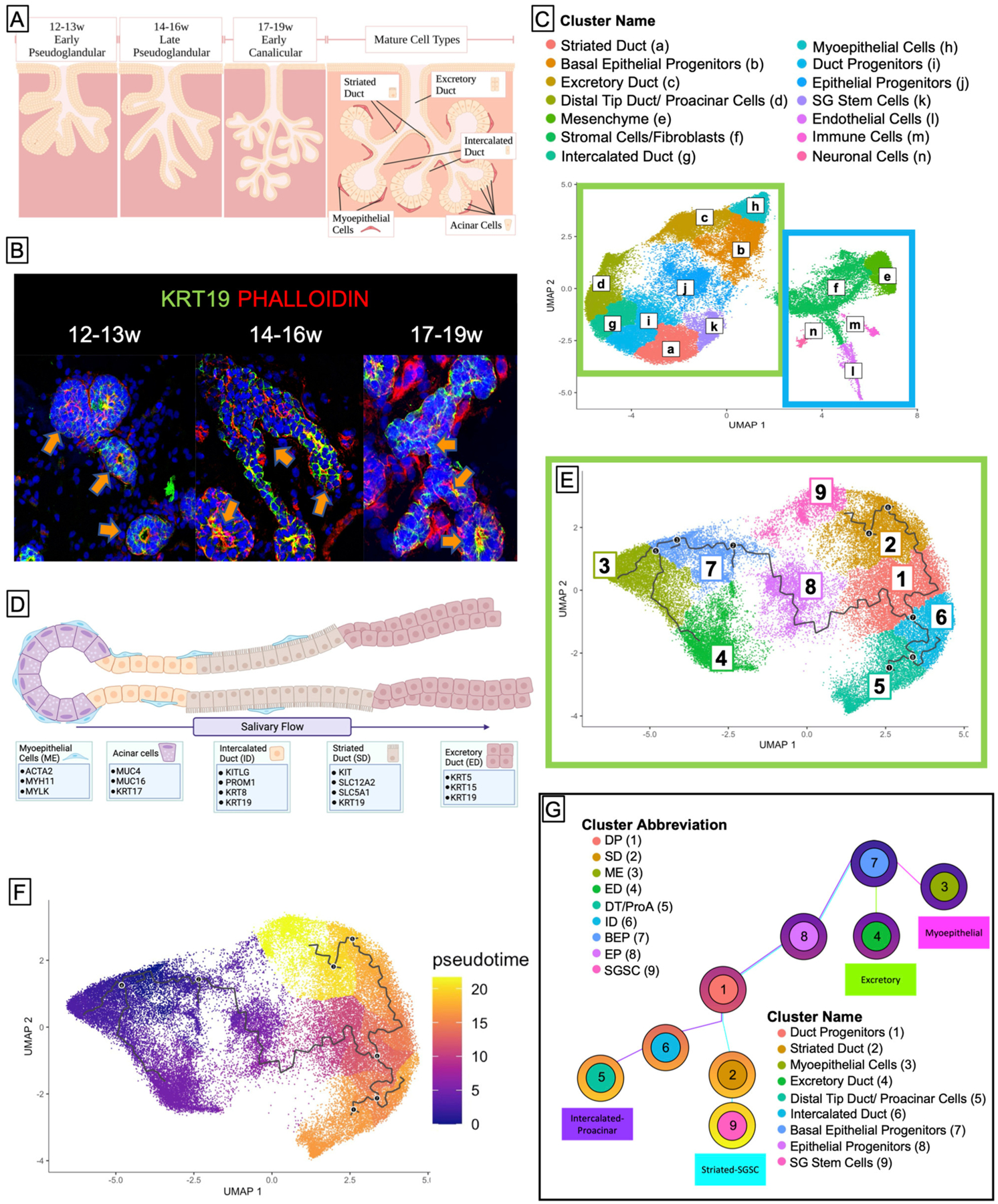
Cell clustering and pseudotime analysis reveal four distinct developmental trajectories for major tissues in the salivary gland. **(A)** Salivary gland development begins in humans at 6 weeks gestation and goes through several stages, the latter of which are the pseudoglandular stage and the canalicular stage. Fully mature salivary glands (not observed until after birth) exhibit 5 distinct types of adult cells: the functional acinar cell, the ID, the SD, the excretory duct, and the myoepithelial cell. Created using BioRender. **(B)** Human fetal tissue at 12 weeks resembles the early pseudoglandular stage, wherein some limited branching can be observed and ducts are mostly non-lumenized or have small lumens. From 14–16w, we see more obvious bifurcations and larger lumens. By 17–19w most ducts exhibit, some kind of lumen, and widespread branching is obvious. **(C)** Saliva flows from the acinar cells through the ID to the SD where it is rendered hypotonic, and then to the excretory duct where it can exit into the mouth. **(D)** Sci-sequencing and unbiased clustering of submandibular human salivary gland tissue from 12 to 19w gestation yielded 16 clusters. The salivary epithelium (green box) is grouped along with an obvious separation from support tissues (blue box). We were able to identify clusters of cells from each ductal type using previously specified markers or functional markers, and though the tissue is yet too immature to have acinar cells, we observed a small subpopulation of cells that expressed genes associated with acinar identity, which we denoted as proacinar cells. **(E)** To facilitate trajectory analysis, we subset the dataset to only include the clusters associated with salivary epithelium [from the green box in **(C)**], resulting in 9 clusters. **(F)** Bioinformatic trajectory prediction and pseudotime analysis suggest that salivary gland development begins with a population of BEPs (Cluster 7) and over several bifurcations, yields distinct trajectories **(G)**.

Salivary gland development begins in humans between 6 and 8 weeks of gestation and continues developing after birth (25). Because of the early stage at which salivary glands develop in humans, the overwhelming majority of what we currently know about salivary gland development has been learned through molecular, cytological, and morphological studies using murine models (26–28). In mice, salivary gland development begins with the invagination of the thickened epithelium into the underlying condensed mesenchyme to yield the initial bud stage (E12.5) (29, 30). Branching morphogenesis and tubulogenesis yield ducts (E13.5–E14.5) (31), and lumenization proceeds through the canalicular stage at E16. Lumenization is a stepwise process whereby lumens form first in the distal end of the main cord and the branch cords, followed by the proximal end of the main cords, and finally the central portion of the main cord (32). Terminal differentiation results in secretory acini (E17.5) that are regulated by nerve stimulation (33). The salivary gland is comprised of a network of branched ducts that terminate in saliva-producing acini. The acini produce primary saliva, which is an isotonic solution containing amylases, mucins, and extracellular fluid. They are connected to intercalated ducts (ID) that receive the primary saliva and serve as a transition between the acini and the functional striated duct (SD), performing minimal ion exchange into the primary saliva. SDs perform a significant amount of active transport to drive ion exchange and water reabsorption to yield hypotonic secondary saliva. This is then transported to the excretory duct, which opens into the oral cavity (Figures 1A–C).

Several major signaling pathways have been identified in salivary gland development in murine models. For example, signaling *via* fibroblast growth factor receptor (FGFR) and epidermal growth factor receptor/erb-B2 Receptor (EGFR/ERBB3) is critical to early salivary gland morphogenesis and development (34–41). Moreover, crosstalk between FGF and wingless/integrated (Wnt) signals has been shown to distinguish between the expanding endbud and the differentiating duct (32), and that Wnt-related transcription factor CP2 like 1 (Tfcp2l1) is required for patterning of salivary gland ducts (42). However, while previous studies have implicated these factors as regulators of early morphogenetic properties like proliferation and branching morphogenesis, thus far, the pathways that regulate individual cell fate choices remain largely unknown. Recently, several groups have undertaken single-cell sequencing in mouse salivary glands at various ages (43–45). These studies underscore the complexity of salivary gland development, highlighting the heterogeneity between glands (44) and identifying critical factors in early development (45, 46). However, human salivary gland signaling pathways and cell-cell interactions that drive individual lineages and cell fate decisions are still to be dissected. To understand the fate and lineage decisions in human salivary gland development, we conducted single-cell sequencing on human fetal salivary tissue.

## MATERIALS AND METHODS

### Tissue Collection

This study is approved by the University of Washington Institutional Review Board (IRB) for the use of human fetal tissues in both the Birth Defects Research Laboratory (CR000000131) and the Ruohola-Baker Laboratory (STUDY00005235). Tissue was collected and dissected as described in Alghadeer et al. (47). Briefly, tissues from 12 to 22 weeks’ gestation were acquired from the University of Washington’s Birth Defects Research Laboratory within 6 h of initial dissection. Salivary glands from 12 weeks gestation and older were isolated from surrounding jaw tissue, and all tissues were snap-frozen in liquid nitrogen-cooled 2-Methylbutane and stored at −80°C until further use. Tissues for sequencing (12–19 weeks) underwent nuclei extraction (48). Tissues that were meant for immunofluorescent staining or RNAScope were embedded in O.C.T. Compound.

### Data Analysis

#### Unbiased Clustering and Cluster Identification

Low-quality reads were removed from each sample by excluding cells with a unique molecular identifier (UMI) <200 and all cells with a high proportion of mitochondrial UMIs. Quality preprocessed datasets were combined to create a single dataset with all age groups and taken through all subsequent analyses. To prevent obfuscation of cell identity in actively cycling cells, we used Seurat to regress out common cell cycle-associated genes from consideration for clustering (49). Cells were clustered using the Monocle3 workflow (50–52). Briefly, data were normalized by size factor, preprocessed using principal component analysis, dimensions were reduced using the UMAP algorithm, and clustered using unsupervised graph-based clustering analysis *via* the Leiden algorithm. In all computational analyses, the statistical significance is assessed by default calculation of a *p*-value, a *q*-value, or a False Discovery Rate set to 0.05 or less; results that fall outside that cutoff are excluded.

Clusters were identified through a combination of literature-derived marker genes from the salivary gland and other glandular epithelium (*via* Panglao DB) and functional characterization of highly expressed genes.

Clusters from the initial analysis were isolated and sub-clustered, following the above procedure with more stringent parameters.

#### Pseudotime Analysis

Pseudotime analysis was performed with the Monocle3 default workflow (50). Briefly, a machine learning technique known as reversed graph embedding is used to “learn” the principal graph and branchpoints that represent the predicted developmental trajectory and embed it back into the graph that represents the single-cell dataset (the cluster plot). Cells are then assigned a pseudotime value based on their projection along the predicted trajectory in relation to the root node and plotted in UMAP with coloring indicative of where a given cell falls in the biological process.

#### Differential Expression Analysis, Top Gene Analysis, and ChEA3 Analysis

To identify differentially expressed genes, we used the R package DE Single (53), which identifies differentially expressed genes between two clusters using a Zero-Inflated Negative Binomial model to faithfully depict significantly expressed genes despite the stochastic nature of transcription at the single-cell level. To ensure the program only returned highly significant results, we set the false discovery rate to <0.05 for all analyses.

To identify the top expressed genes per cluster, we used Monocle3’s top markers function. This function classifies genes based on multiple parameters including expression level as well as how unique its expression is to a given cluster and its pseudo R^*2*^ value and assigns them a “marker score”. We further asked the program to only consider genes that were expressed in at least 25% of the cells and asked to return the top 300 ranked genes per cluster.

The ChEA3 (54) is an accessible programming interface that allows users to submit gene sets for analysis and compares them to six separate databases of various types of ChIP experiments to identify the most likely active transcription factors yielding a given list. We conducted this analysis on both the top 300 genes per cluster and the gene list output from DE single to identify important transcription factors driving both cell identity and cell fate change along a trajectory.

#### Gene Module Analysis

To identify groups of genes that are similarly regulated, we used Monocle3’s gene module analysis. This vignette runs UMAP on the genes as opposed to the cells, placing them into groups for Louvain community analysis. The output was visualized both as a heatmap representing how enriched a given module is in each cluster and plotted over the UMAP to visualize whether a module was shared across partitions or across clusters, or whether it was consigned to a specific partition or region of a cluster.

#### FeatureScatter Analysis for Gene Co-expression

To demonstrate co-expression (or lack thereof) of genes in single cells within a cluster, we used Seurat’s FeatureScatter vignette (55) which allows any quantifiable parameter that can be retrieved from the matrix columns to be plotted in a scatter plot against any other feature. To achieve this, we converted our Monocle3 cell data set (cds) file to a Seurat Object. Since our feature of interest was gene expression, we set Feature1 to one gene and Feature 2 to a second gene. The output was a scatter plot where each axis was the normalized expression of one of the genes, and each dot represents a single cell.

### Immunofluorescent Staining and Confocal Imaging

Salivary glands intended for immunofluorescent staining were snap-frozen as described above and embedded in O.C.T. compound (Tissue-Tek, #4583). Using a Leica CM1850 Cryostat, embedded tissues were cryosectioned into 10 μm cuts and mounted on SuperFrost Plus slides (Fisher Scientific #12-550-15). Prior to staining, slides were washed in 1x PBS for 5 min to remove O.C.T compound, then overlaid with 4% paraformaldehyde for 10 min to fix. After fixation, slides were washed three times for 5 min, then overlaid in a blocking solution containing 5% bovine serum albumin, 1% normal goat serum, and 0.1% Triton-X and left to incubate at room temperature for 90 min. After blocking, slides were overlaid with primary antibody (Table 1) diluted in blocking solution and placed in a humidity box at 4°C overnight. The next day, slides were washed three times in 1x PBS for 5 min and overlaid with AlexaFluor-conjugated secondary antibodies (Life Technologies, 1:200) or preconjugated primary antibodies diluted in blocking solution according to manufacturer recommendation and incubated at room temperature for 75 min. Slides were then washed four times for 5 min and overlaid with DAPI diluted in 1x PBS and incubated at room temperature for 20 min. Slides were washed a final time in 1x PBS for 15 min, then mounted with Vectashield Hardest Antifade Mounting Medium (Vector Laboratories #H-1400) and allowed to sit overnight in the dark at 4°C. After staining, slides were stored at 4°C in the dark. Slides were imaged on an inverted Nikon Eclipse Ti inverted microscope equipped with an A1R point scanning confocal system with alkali photomultiplier tubes for blue and far-red detection, and GaAsP photomultiplier tubes for green and red detection. Images were taken at 40×–60× magnification and 1,024 × 1,024 resolution and processed using NIS Elements Advanced Research imaging software (Version 5.11.01) and Fiji ImageJ (56) (Version 2.0.0-rc-69/1.52p).

### RNAScope Fluorescent *in situ* Hybridization

Freshly cut human fetal tissue sections were prepared and stained according to the manufacturer’s instructions for RNAScope HiPlex Fluorescent *in situ* Hybridization (ACDBio). Briefly, tissues were fixed in fresh 4% paraformaldehyde for 60 min at room temperature, dehydrated with increasing concentrations of ethanol (50, 70, 100%), and dried. A hydrophobic barrier was created around each tissue section and slides were treated with protease for 30 min at room temperature. Sections were then overlaid with a probe and incubated for 2 h at 40°C. Following hybridization, slides were stored in 5x SSC buffer at room temperature overnight. On the next day, Amp1, Amp2, and Amp3 were hybridized in succession at 40°C for 30 min, with two 2-min washes with 1x Wash Buffer after each hybridization. Fluorophores were hybridized for 15 min at 40° C, slides washed twice with 1x Wash Buffer, overlaid with DAPI for 30 s, then mounted with the Fluoromount-G mounting medium (Invitrogen), and allowed to cure overnight at 4°C. Slides were imaged with a Nikon W1 Yokogawa Spinning Disk Confocal microscope equipped with a 40x water immersion lens and an Andor iXon 888 EMCCD camera. Images were processed with Fiji ImageJ (56) (Version 2.0.0-rc-69/1.52p).

## RESULTS

### Cell Clustering and Pseudotime Analysis Reveal Three Distinct Developmental Trajectories for Major Tissues in the Salivary Gland

In order to identify individual cell populations in the developing human fetal salivary gland development, we performed single-cell combinatorial indexing RNA sequencing (sciRNAseq) (57) on human fetal submandibular salivary glands from three developmental timepoints: 12–13 weeks’ gestation, 14–16 weeks’ gestation, and 17–19 weeks’ gestation (Figures 1A,B). Unbiased clustering using Monocle3 resulted in 14 unique clusters (Figure 1D) that were identifiable by molecular and functional markers (Supplementary Figures 1A–C; Figure 1C). Cell clusters partitioned clearly along epithelial and non-epithelial lines (Figure 1D, green/blue boxes). The large left island of clusters (green box) is comprised of the epithelial portions of the salivary gland, which means that the duct types and myoepithelial tissues, while the right side (blue box) is comprised of support tissues of the salivary gland including the mesenchyme that surrounds the ducts, fibroblasts, and stromal cells, as well as endothelial, immune, and neuronal type cells.

To examine the epithelial developmental trajectory, we isolated the epithelial clusters and conducted trajectory (Figure 1E) and pseudotime analysis (Figure 1F), which predict the likely developmental trajectory of a given cell set through a biological process. These data predict that the earlier progenitor types (clusters 7 and 1) give rise to more mature cell types that appear at later developmental timepoints (clusters 2–6). This developmental prediction suggests that the major cells in the salivary epithelium arise through three distinct trajectories (Figure 1G), beginning with basal epithleial progenitors (BEPs) in cluster 7. BEPs give rise to myoepithelium (cluster 3) and excretory duct (cluster 4), and, through a transitional cluster, to duct progenitors (cluster 1). Duct progenitors (DPs) give rise to either ID (cluster 6) or SD (cluster 2). Finally, ID ultimately can give rise to the proacinar cells *via* distal tip duct reorganization (cluster 5), and some of the SD yields a stem cell-like population (cluster 9).

### Basal Epithelial Progenitors Give Rise to Excretory Duct and Myoepithelial Cells With Stage-Specific Functions

**FIGURE 2 |:**
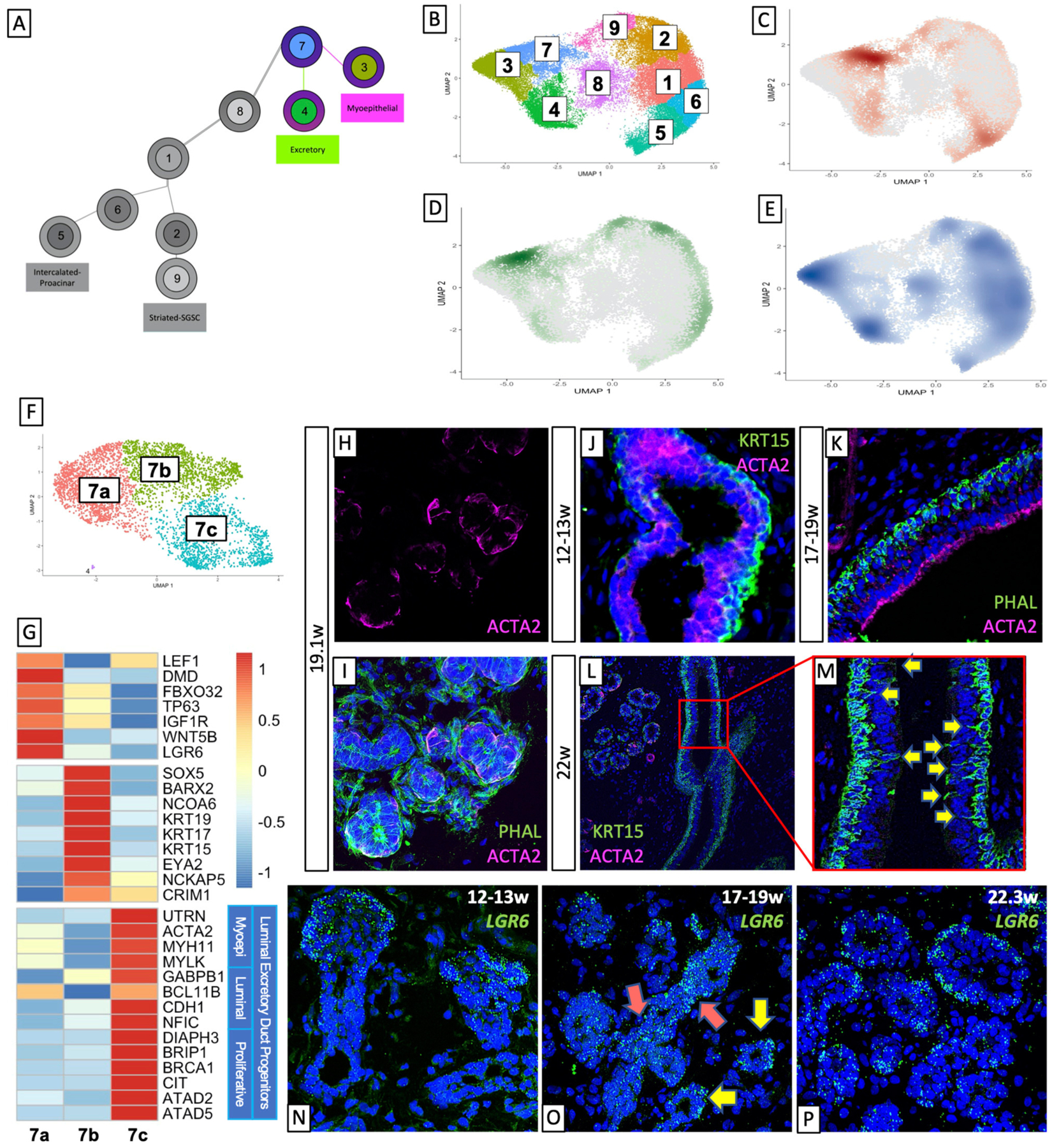
Basal epithelial progenitors give rise to excretory duct and different populations of myoepithelial cells. **(A)** Early BEPs give rise to Myoepithelial cells and Excretory Duct. Density plotted by age **(C-E)** in the salivary epithelium **(B)** showed that early cells are overrepresented in the BEPs and at 12–13 weeks **(C)**, are moving toward the excretory duct. At 14–16 weeks **(D)** the bias of cells shifts toward the myoepithelial cluster, and by 17–19 weeks **(E)** most cells have moved out of BEPs and occupied their respective mature populations. **(F)** Subsetting BEPs yields 3 clusters. **(G)** Top gene expression shows a distinct transcriptional expression of each cluster, with 7a expressing myoepithelial progenitor markers, 7b expressing basal duct markers, and 7c expressing groups of genes with distinct functional associations. **(H,I)** Human fetal tissue at 19.1w shows an expression of ACTA2 surrounding traditional ductal types. **(J,K)** Human fetal excretory duct at 12–13w **(J)** and 17–19w **(K)** show basal expression of KRT15 and luminal expression of ACTA2. **(L)** Human fetal salivary glands at 22w only exhibit ACTA2 expression around intercalated/straited duct types, but not lining the luminal excretory duct expression of ACTA2. **(M)** KRT15 in the basal excretory duct shows that basal duct cells exhibit membrane protrusions toward the luminal side. **(N-P)** *LGR6* expression at 12–13w **(N)** and 17–19w **(O)** show the presence of BEPs in non-lumenized ducts. 17–19w and 22.3w **(P)** samples show expression of *LGR6* around the outer edge of lumenized ducts, in line with myoepithelial cell localization.

According to our predicted trajectory, BEP (cluster 7) give rise to the excretory duct (ED, cluster 4) and myoepithelial tissue (ME, cluster 3, Figure 2A). Temporal density analysis of the UMAP (Figures 2B–E) indicated that BEP present at the 12–13w timepoint tended toward the excretory duct (Figure 2C) while those present at 14–16w tended toward ME (Figure 2D), leaving most cells in their respective populations by 17–19w (Figure 2E). The BEP cluster is subset into three distinct clusters (Figure 2F), wherein the temporal distinction persists (Supplementary Figures 2A–C), with each of the three clusters hosting the majority of cells at a given timepoint. Molecular analysis of the clusters (Figure 2G) revealed that highly expressed genes in cluster 7a (pink), which is primarily comprised of cells aged 14–16w, were common myoepithelial progenitor markers (58–61), while cluster 7b (green) was comprised primarily of cells of 12–13w and predominantly expressed markers for basal duct epithelium (62, 63). Cluster 7c (turquoise) was less clear, as it contained cells from all timepoints, and, intriguingly, expressed high levels of mature myoepithelial markers like UTRN, ACTA2, and MYH11, but also expressed luminal epithelial markers (64–69) and several genes that drive proliferation (70–74). This analysis suggests that these early BEPs consist of expected myoepithelial progenitors (7a) and basal excretory duct progenitors (7b), but also yield a proliferative luminal duct progenitor that also expresses mature myoepithelial markers (7c).

#### TSPEAR Is Enriched in the Early Excretory Duct

The ED is the only stratified (multilayered) duct in the developed salivary gland. It was easily identifiable as expressing high levels of basal keratins (KRT5, KRT15, Figures 2L,M; Supplementary Figures 1A,B) and gene ontology analysis for top expressed genes within the cluster returned terms associated with salivary gland development, epithelial cell polarity, and migration, and tube formation, among others (Supplementary Figure 2F). One of the more intriguing finds within this cluster (cluster4) was the very high and unique expression of a gene called TSPEAR, a thrombospondin-type laminin. A recent study (75) found TSPEAR to be mutated in a familial syndrome in which affected individuals exhibited hypodontia and hypotrichosis and misregulated Notch signals. Interestingly, another protein that is usually found in epithelial progenitor layers and in some cases regulating Notch ligands, TP63, is also highly expressed in the excretory duct cluster (76), and Notch signaling appears as a significantly enriched GO term (Supplementary Figure 2F). In mammary development, Notch signaling has been demonstrated to drive luminal identity (77). Further studies are required to determine whether TSPEAR and Notch may also promote luminal identity in developing salivary excretory duct.

#### ACTA2+ Cells Occupy Two Temporally Distinct Locations During Development

In mature salivary glands, ACTA2+ MEs are localized around the acini and some of the upper IDs and SDs (Figure 1C). We observe this localization only in more mature fetal tissues (19–22w, Figures 2H–L). However, our transcriptomic analysis suggests that ACTA2+ myoepithelial cells are present beginning at 14 weeks, and BEP subset cluster 7c expresses a low level of ACTA2 (and other myoepithelial markers). To further investigate this phenomenon *in vivo*, we stained fetal submandibular glands with basal epithelial marker KRT15 and myoepithelial ACTA2 (Figures 2J–L). We observed that by 12–13w, KRT15 is beginning to be expressed in the cells on the outer surface of the developing duct while a low level of ACTA2 expression was observed in cells located toward the luminal side (Figure 2J). By 17–19w, the delineation between basal and luminal duct is complete and ACTA2 is clearly expressed on the luminal side (Figure 2K). These data suggest that the early ACTA2+ cells may represent a distinct population with a function unique from the later acinar- and duct-associated myoepithelial cells. Previous studies (78) have shown that mature human submandibular salivary glands do not exhibit ACTA2 expression in their excretory duct, suggesting that ACTA2+ cells in the luminal ED represent a transient population of cells during early differentiation. Consistent with this observation, in 22w human fetal tissue (Figure 2L), though we still observe the basal expression of KRT15, we no longer observe ACTA2+ luminal excretory duct; we only observe ACTA2 expression surrounding non-excretory type ductal tissues (Figures 2I,L). Subsetting the myoepithelial cluster by age demonstrates that cells from 12 to 13w are, in addition to far fewer in number than in other timepoints, indeed more immature. They exhibit higher expression of myoepithelial progenitor markers. As cells in this cluster age, they lose expression of the myoepithelial progenitor genes and increase the expression of mature myoepithelial markers like MYH11, MYLK, ACTA2, and others (Supplementary Figure 2D). Gene ontology analysis is consistent with this observation, with 12–13w samples engendering early developmental terms including epithelial development and polarity, and tissue morphogenesis, and later timepoints yielding terms with epithelial tissue differentiation and involuntary muscle activity. Top genes that appear in all three age groups (79), as in the 12–13w group by itself, yield terms of broader epithelial development and polarity, while those expressed only in the older tissues (80) drive actin cytoskeletal reorganization and involuntary muscle contraction. Interestingly, this indicates that even the ME observed in the excretory duct at 17–19w represent a more mature myoepithelial population. The change in maturity can be visualized with a combined heatmap of the top 300 genes per age group, showing the transition from more immature markers in 12–13w toward more mature markers at 17–19w (Supplementary Figure 2E). Interestingly, this indicates that even the myoepithelial cells observed in the excretory duct at 17–19w represent a more mature epithelial population. Curiously, by 22 weeks gestation, we also observe that KRT15+ basal cells are beginning to exhibit filamentous protrusions toward the lumen (Figure 2M, yellow arrows). Previous studies in the tracheal surface airway (81) found that in mice, ACTA2+ myoepithelial cells lose ACTA2 expression once they have differentiated into other subtypes, and mammary studies have shown that myoepithelial cells secrete factors that are critical for lineage differentiation (82–84), suggesting that in salivary gland development, this early myoepithelial population is a transient population that may serve to guide early fate choices in the absence of a more complex and/or defined environment.

#### LGR6 Is Expressed in the Early Salivary Gland (BEP and ME)

Leucine-rich repeat-containing G-protein-coupled receptor (LGR) family genes are well-characterized as adult stem cell markers and are often found misregulated in epithelial cancers (85). LGR6 is known to mediate Wnt signaling through R-spondins (86). In mammary glands, LGR6+ cells represent a population of progenitor-type cells that contribute to luminal cell expansion (87). In sweat glands, LGR6+ cells marked a label-retaining population of cells that were also ACTA2+ (88). We show through bioinformatic analysis that LGR6 expression was enriched in ME cells (Supplementary Figure 2F, pink arrow), and in BEP (Supplementary Figure 2F, purple arrow). In the BEP subset, while we observed the majority of LGR6 expression in cluster 7a, there is also an expression of LGR6 localized to cluster 7c (Supplementary Figure 2G). To validate these results, we performed RNAScope *in situ* hybridization for *LGR6* (Figures 2N–P). Expression of *LGR6* was abundant at 12–13w, validating the presence of the BEP population in early non-lumenized ducts (Figure 2N). In 17–19w timepoints prior to the appearance of myoepithelial cells around ducts (Figure 2O), we observed more widespread expression of *LGR6*, but here we observed two patterns. First, in ducts with smaller lumen, we observed a more ubiquitous expression of *LGR6* (Figure 2O, pink arrows), but in ducts that already possessed an obvious lumen, we observed that *LGR6* expression became relegated to the basal side of the duct (Figure 2O, yellow arrows), reminiscent of the localization of forthcoming myoepithelial cells. At 23w (Figure 2P), we observed almost all *LGR6* expression in cells on the basal side of the duct, at the same locations where we observe ACTA2 expression (Figures 2I,L). Myoepithelial cells in various epithelial tissues have been shown to exhibit a level of plasticity (61, 89, 90) consistent with their ability to participate in injury repair, and injury repair studies in adult mouse airway epithelium discovered that ACTA2+/LGR6+ cells that can conduct targeted repair after injury (91). Considering this, our data also support the notion that later LGR6+/ACTA2+ salivary myoepithelial cells, in addition to their traditional role as a contractile cell that moves saliva out of the acinar tissue, may harbor a level of plasticity that can allow them to participate in repair, thereby representing a novel source of progenitor-like that will require further exploration.

### Bifurcation of Ductal Progenitors Correlates With Differential Regulation of Secreted Signals and Cell–Cell Interaction

Trajectory analysis suggests that DPs (Cluster 1) are derived from BEP through a transitional group (Cluster 8, Epithelial Progenitors, EP, Figure 1G). A comparison of highly expressed genes from both DP and BEP showed that these genes were also enriched in EP (Supplementary Figures 2H,I), consistent with that observed in Supplementary Figure 1A. Differential expression analysis of EP did not reveal independent expression of DP and BEP-associated genes; instead, top marker analysis showed that both clusters expressed high levels of genes associated with epithelial progenitor type cells (92–95) and salivary epithelium (96, 97), suggesting this represents a transitional population in the process of changing developmental programming.

**FIGURE 3 |:**
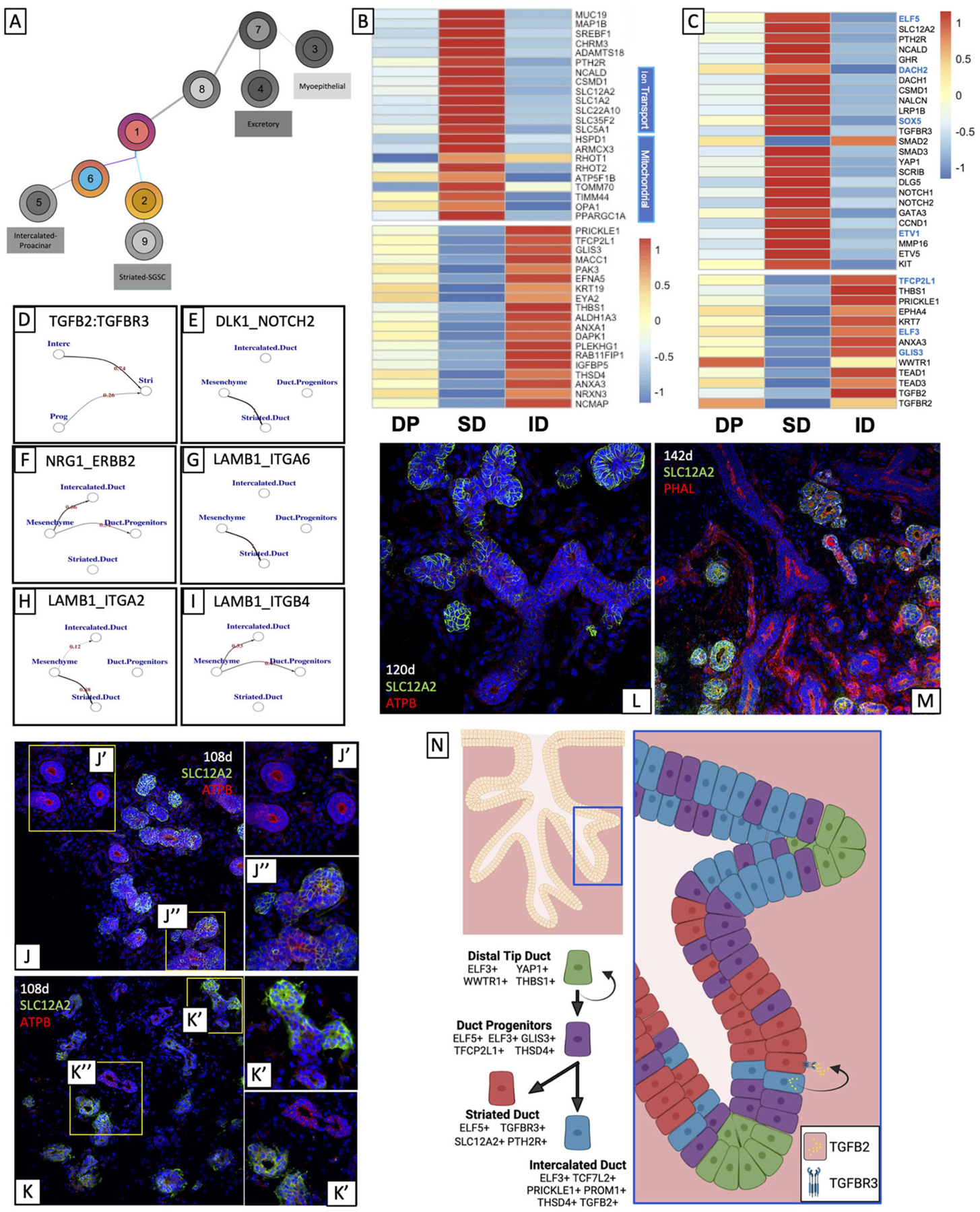
Bifurcation of ductal progenitors relies on differential regulation of secreted signals and cell-cell interaction. **(A)** DPs split into IDs and SDs. **(B)** Top gene expression analysis of striated and IDs show that DPs share transcriptional overlap with both striated and ID, but much more so with ID. **(C)** Differential expression and ChEA3 analysis and ligand-receptor analysis **(D-F)** suggest that TGFβ signaling may promote striated identity while Wnt promotes intercalated identity. ECM-cell interaction analysis **(G-I)** suggests that interaction between laminin and different integrins may also contribute to this bifurcation. Top marker analysis suggested that SLC12A2 was almost uniquely expressed by SD. We observed expression consistent with this in fetal tissue **(J-M)**. Analysis suggests that ELF3 and ELF5 mark intercalated and SDs respectively, and that TGFB2 from ID helps to drive TGFβ signaling in SD **(N)**. Created using BioRender.

Pseudotime analysis suggests that DP is split into two types of ducts: SD and ID (Figure 3A). Identification of highly expressed genes in ID and SD (Figure 3B; Supplementary Figure 1A), as well as gene ontology analysis showed that highly expressed genes in the ID (Supplementary Figure 3G) were associated with basic duct development including epithelium development, structure morphogenesis, and ECM organization, while highly expressed genes in the SD (Figure 3B; Supplementary Figure 3F) were more functionally oriented: gene ontology terms for ion and vesicle transport, mitochondrial function, and the anti-microbial response was highly ranked.

Transcriptional analysis of both ID and SD (Figures 3B,C; Supplementary Figures 3A–E) showed that ID more closely resembles DP, suggesting that ID is the “default” duct type while SD will require input signaling to drive an expression program that drives SD identity. ID also exhibits high levels of DAPK1, a calcium calmodulin-dependent serine/threonine kinase that drives mitophagy (80, 98), and RAB11FIP1, which promotes endosomal recycling (99), inverse cellular needs to those observed in SD, which utilize mitobiogenesis and exocytosis (100).

#### Striated-Intercalated Bifurcation Involves Differentially Regulated Wnt, TGFβ and Notch Signaling

DP and ID express high levels of several genes that regulate Wnt signaling (Supplementary Figure 3D), the most prominent of which is PRICKLE1, a protein that activates non-canonical Wnt signaling to drive planar cell polarity (101) and has also been shown to have context-dependent agonistic and antagonistic effects on canonical Wnt signaling (102). ChEA3 Transcription Faction prediction (Supplementary Figure 3E; Figure 3C, blue genes) based on the differentially upregulated genes in the intercalated cluster predicted the activity of Wnt-related transcription factors with the significant expression: the CTNNB1 transcriptional cofactor TCF7L2, and the transcription factor TFCP2L1. Both transcription factors are responsible for the expression of multiple differentially expressed genes in ID cells (Figure 3C, black genes below corresponding blue genes), but most interestingly, they are predicted to drive the expression of ID-specific genes THBS1, PRICKLE1, and KRT7, suggesting that Wnt signaling may be involved in promoting the ID identity.

Ligand-receptor analysis on the salivary epithelium and support tissues including mesenchyme suggests that crosstalk takes place between ID and SD. Using a ligand-receptor analysis program called talklr (103), which identifies significant interactions between cell clusters, we found a significant interaction between TGFB2-expressing ID and TGFBR3-expressing SD (Figure 3D). Interestingly, although ID expresses high levels of TGFB2 and one of its receptors, TGFBR2, it also expresses high levels of a gene called THSD4 which has been shown to repress TGFβ (104), suggesting that TGFβ signaling is not autocrine and can be inhibited to maintain intercalated identity. Moreover, ID also expresses several other secreted factors in addition to TGFB2 including ANXA1 and THBS1 that can promote TGFβ signaling through TGFBR2 and TGFBR3 (105–108), suggesting that secreted factors from ID can drive striated identity in neighboring cells that express TGFβ receptor. Furthermore, KEGG analysis of ID enriched genes supports this, showing that while ID express secreted factors, they do not exhibit intracellular indicators of active TGFβ signaling (Supplementary Figure 3H). The analysis also suggested that SD exhibited enrichment of Notch signaling mediators. SD exhibited enriched expression of NOTCH1 and NOTCH2, and talklr suggested that there was a significant interaction between NOTCH2 in the SD and Notch ligand DLK1 expressed by the mesenchyme (Figure 3E). Moreover, NOTCH1 and NOTCH2 are both transcriptional targets of SOX5. In some contexts, TGFβ has been shown to act synergistically with Notch (109, 110), and SOX5 has been shown to be a DNA binding co-factor for Smads (111). We, therefore, hypothesize that after TGFβ activation occurs, SDs may maintain their identity by synergistic TGFβ and Notch regulation.

E74-like ETS Transcription Factor 3 and 5 (ELF3 and ELF5) are two of a family of epithelium-specific ETS transcription factors defined by their highly conserved ETS DNA binding domain (112). ELF3 was enriched in ID and DP, while ELF5 was enriched in SD, and ChEA3 analysis predicted them as responsible for several genes preferentially expressed by each cluster, suggesting that these two factors can contribute to the ID-SD bifurcation. ELF3, which is predicted to drive the expression of highly expressed ID genes ANXA3, TGFB2, and duct marker KRT8, has been shown to drive ligand-independent transactivation of CTNNB1 in cancer models (113, 114).

#### Sodium-Potassium Transporter SLC12A2, an ELF5 Target, Marks Developing Striated Ducts

Among SD-specific genes attributed to ELF5, we identified SLC12A2, a sodium chloride transporter, as the most highly expressed gene in that cluster, and almost exclusive to SD. SLC12A2 has been shown in mouse salivary glands to be expressed in SD during development (43, 115) and is also in secretory acini in mature glands (116, 117). To validate this ELF5 target as an SD-specific marker in developing human salivary glands, we located SLC12A2 in human fetal salivary glands using immunocytochemistry (Figures 3J–M). In a manner consistent with sci-Seq clustering, several of the larger, more numerous SDs expressed SLC12A2 (Figure 3J”), while some of the smaller IDs did not (Figure 3J’). We can observe that in ducts with multiple branches, SLC12A2 is distinctly expressed in specific branches, (Figure 3K’), representative of the bifurcation of IDs and SDs in development. We observe this distinction between SLC12A2+ SDs and SLC12A2- IDs through later timepoints (Figures 3L,M). Interestingly, though ELF5 has previously been shown to drive TGFβ signaling it is unclear whether TGFβ likewise drives ELF5 expression. Interestingly, traumatic brain injury studies (118) have shown that antagonizing TGFβ expression also diminished SLC12A2 expression, suggesting that SLC12A2 expression in human fetal SD might also be driven by TGFβ signals from the ID, and it will be interesting to evaluate this in the future in in vitro salivary gland models.

#### Cell-ECM Interactions May Be Important to Facilitate SD/ID Bifurcation

The mesenchyme expresses a significant amount of laminin (LAMB1), which binds to various integrins (119). Talklr suggests that LAMB1 interacts with different integrins to preferentially drive either SD or ID (Figures 3G–I). LAMB1 interactions with ITGA6 (Figure 3G) and ITGA2 (Figure 3H) are predicted to drive SD, while its interaction with ITGB4 (Figure 3I) favors ID identity. Classically, laminin-integrin interactions drive epithelial cell polarity and migration (120, 121), and mouse studies in the salivary gland have shown that LAMB1-ITGA6 interactions are critical for branching morphogenesis in early developmental (122), however, it is unclear how laminin-integrin signaling plays into this later-stage bifurcation. Several studies have shown that integrin-laminin signaling can affect the expression of functional genes downstream (123–125). One interaction of particular interest found that ITGA6 was able to drive Notch signaling in endothelial cells during angiogenesis (126), consistent with our prediction that Notch signaling is enriched in the SD cluster, suggesting laminin-integrin interactions can bolster intracellular signals that can bias DP toward a striated lineage, combining both cell-cell mediated signaling with secreted signaling.

Taken together, our data suggest that DPs are ELF3+ and ELF5+ ductal cells that bifurcate into ELF5+/SLC12A2+ SD and ELF3+/SLC12A2- ID. It also suggests that TGFB2 produced by ID drives TGFβ signaling in SD and may help to drive this bifurcation (Figure 3N).

### Straited Duct Gives Rise to a Population of Stem Cell-Like Cells Late in Development

**FIGURE 4 |:**
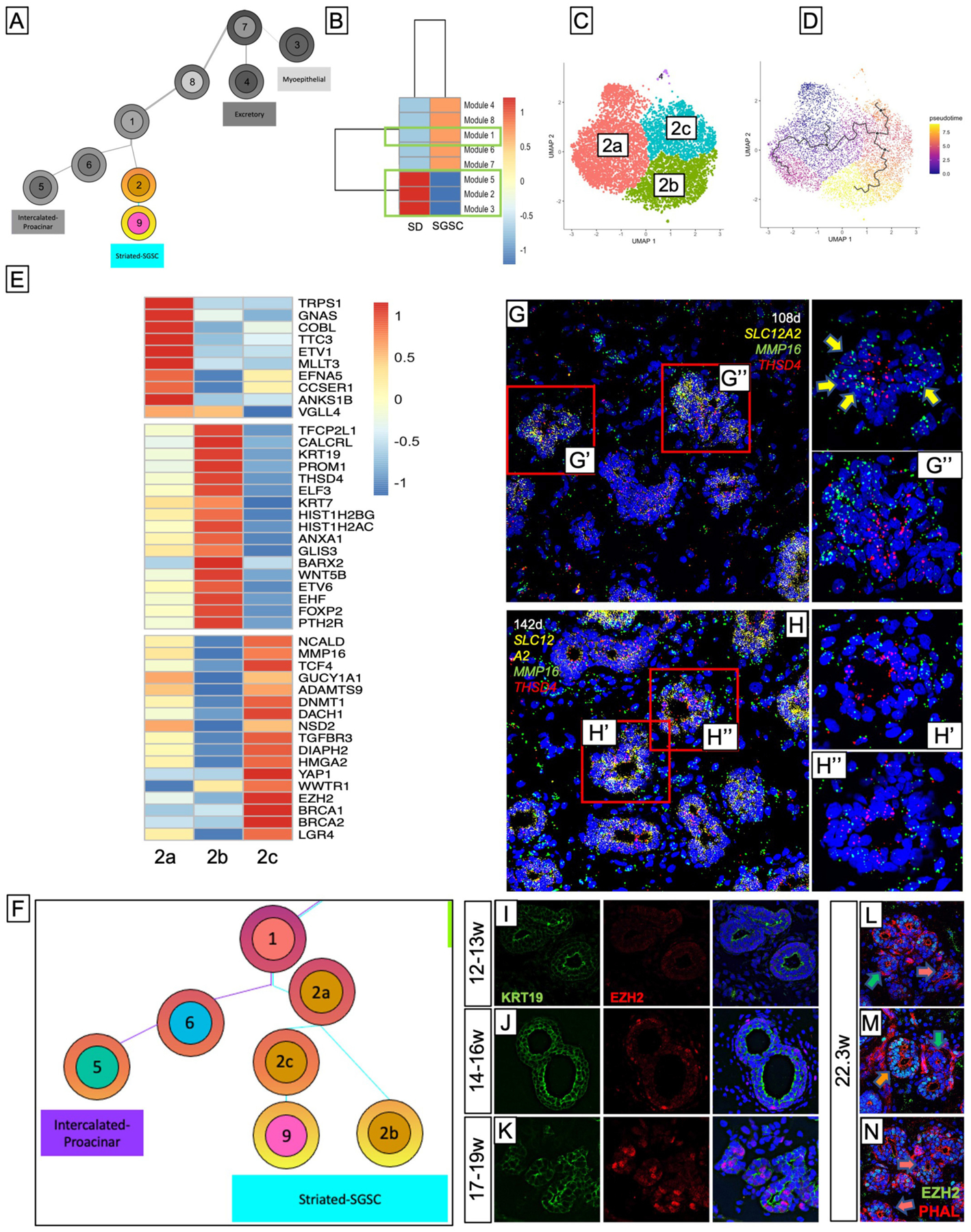
Striated duct gives rise to a population of stem cell-like cells late in development. **(A)** SD gives rise to a subsequent cluster of stem cell-like cells. **(B)** Module analysis revealed that SD has three distinct modules of co-regulated genes. In conjunction with this, SD subsets into three distinct clusters **(C)**, where 2a gives rise to 2b and 2c. **(D)** Transcriptional analysis shows distinct transcriptional patterns for each cluster, with 2a showing transcriptional overlap with both 2b and 2c. **(E)** Cluster 2b expresses genes in line with duct identity while Cluster 2c expresses genes associated with stem cell-like function. **(F)** Analysis suggests that rather than DPs directly giving rise to SD, it gives rise to an early SD-like precursor that can give rise to either SD or the subsequent population. **(G,H)** RNA Scope *in situ* hybridization shows that at 17–19w SLC12A2+ SDs have cells that co-express stem cell-expressed MMP16 and SD-expressed THSD4, marking cluster 2a, while later tissues showed more distinct expression of each of these markers. Using EZH2 to mark stem cells, we found that EZH2 enriched cells were unobservable at12–13w **(I)**, but become increasingly abundant after 14w **(J,K)**. Throughout the 22w salivary gland, we observe overall more abundant cells within the ducts that are enriched for EZH2, but we still observe ducts that do not exhibit EZH2 enrichment [**(L,M)**, green arrows] which, given their size and localization, are likely IDs, ducts that look similar to those observed at earlier ages with select cells enriched for EZH2 [**(L,N)**, coral arrows], and, much more rarely, ducts in which almost all of the cells exhibit enriched EZH2 [**(M)**, orange arrow].

Trajectory and pseudotime analysis (Figure 4A) suggests that SD further gives rise to a population of cells later in development that share similar, albeit reduced, expression of several genes from the striated population, but acquired expression of a host of genes associated with stem cell-like identity, including proliferation, self-renewal, and DNA repair (Supplementary Figure 4F). Because it is paradoxical that all the SD cells would give rise to this stem cell-like population, we sought to understand how some of the SD cells change fate to a stem cell-like identity. To do this, we performed module analysis (Figure 4B; Supplementary Figures 4D,E), which identifies groups (modules) of co-regulated genes that vary in some interesting way across clusters. This analysis revealed a total of 8 modules, but the majority of the top expressed genes in the two clusters were contained in modules 1–3 and 5 (Figure 4B, green boxes). Interestingly, this analysis showed that the striated cluster has three distinct modules, one of which is enriched at the border the striated cluster shares with the stem cell-like cluster (Module 5), suggesting that those cells may represent transitional cells. To further investigate this, we subset the SD with more stringent parameters. Subsetting yielded 3 clusters (Figure 4C) where cluster 2a was populated by cells from all-time points and clusters 2b and 2c appeared at later timepoints (Supplementary Figures 4A–C), consistent with pseudotime analysis that suggests cells from cluster 2a yield either cluster 2b or 2c (Figure 4D). Top gene expression analysis (Figure 4E) showed that clusters 2b and 2c have distinct gene expression profiles, while a significant portion of the gene expression in cluster 2a overlapped with both clusters 2b and 2c, consistent with a progenitor-type cell. Genes associated with Golgi to membrane transport, sodium/ion transport, and epithelial cell development are expressed in all 3 subset clusters, highlighting their shared SD identity. However, cluster 2b exhibits a higher expression of genes associated with duct identity, while cluster 2c exhibits a higher expression of several genes associated with epigenetic modification (127, 128), and stem cell niche maintenance (129, 130). Additionally, it expresses increased levels of TGFBR3, which is expressed primarily by other progenitor groups and the stem cell-like population. Transcription factor prediction with ChEA3 suggested that in Cluster 2b, TFCP2L1 (131) and epithelium-specific ETS transcription factors EHF and ELF5 (112) were responsible for the expression of many genes driving ductal identity, including KRT19, KRT7, mTOR complex regulator DEPTOR, PROM1, and adherins junction regulators PLEKHA7 and AFDN. On the other hand, top-ranked transcription factor predictions for Cluster 2c included DNMT1 and HMGA2, both transcriptional cofactors and epigenetics modifiers which have been associated with progenitor maintenance and self-renewal (132–135). Taken together, this suggests that the DP gives rise to a type of broader SD progenitor group (2a) that further yields two types of SD: one type that maintains its identity as a duct and ultimately matures into proper SD (group 2b), and another (group 2c) that is capable of yielding a previously undescribed population of stem cell-like cells that maintain, albeit reduced, some transcriptional properties of SD while also acquiring stem cell characteristics like progenitor maintenance and self-renewal (Figure 4F).

To locate cluster 2 subsets within the developing tissue, we performed RNAScope *in situ* hybridization. We sought to identify cells co-expressing *SLC12A2*, which marks all SD and either *THSD4* to mark duct-bound cells (cluster 2b), or *MMP16* to mark stem cell-bound cells (cluster 2c) (Figures 4G,H). Consistent with IF stains of SLC12A2 (Figures 3J–M), we found *SLC12A2* was highly enriched in SDs, but not IDs. At the 108d timepoint (17w), we observe that some of the *SLC12A2*+ cells express both *MMP16* and *THSD4* (Figure 4G’, yellow arrows), suggesting these to be the precursor cells (cluster 2a), but we observe far more cells with distinctive expression of either *MMP16* or *THSD4* within the same duct (Figure 4G”). Additionally, we observed that the *THSD4*+ cells were more often toward the apical side of the duct while the *MMP16*+ cells were more often toward to basal side, suggesting that this developmental distinction occurs or at least begins with the help of some form of duct polarity. At 22w (Figure 4H), we observe far fewer *THSD4/MMP16* double-positive cells and more distinction between the two populations. Moreover, although the stem cell cluster appears among the latest in the developmental trajectory (Supplementary Figure 4G), proliferation scoring shows that they maintain a very proliferative identity (Supplementary Figure 4H), suggesting that at later stages of development, SD stops giving rise to the stem cell precursor group and already-specified stem cells propagate *via* self-renewal.

Transcriptomic analysis revealed EZH2 as highly enriched in cluster 9 (Supplementary Figure 4I, pink arrow). Using EZH2 as a marker, we observed that although all cells express some level of EZH2, which can be expected in an actively developing tissue, there were certain cells within the ducts that had unmistakably enriched EZH2 expression compared to surrounding cells within the same duct. These cells are almost undetectable at 12–13w (Figure 4I) and are extremely rare in the 14–16w timepoints (Figure 4J) but become more abundant in the 17–19w timepoint (Figure 4K). This population persists at least through 22 weeks’ gestation (Figures 4L–N).

### Bioinformatic Analysis Introduces New Potential Regulators of Acinar Differentiation

**FIGURE 5 |:**
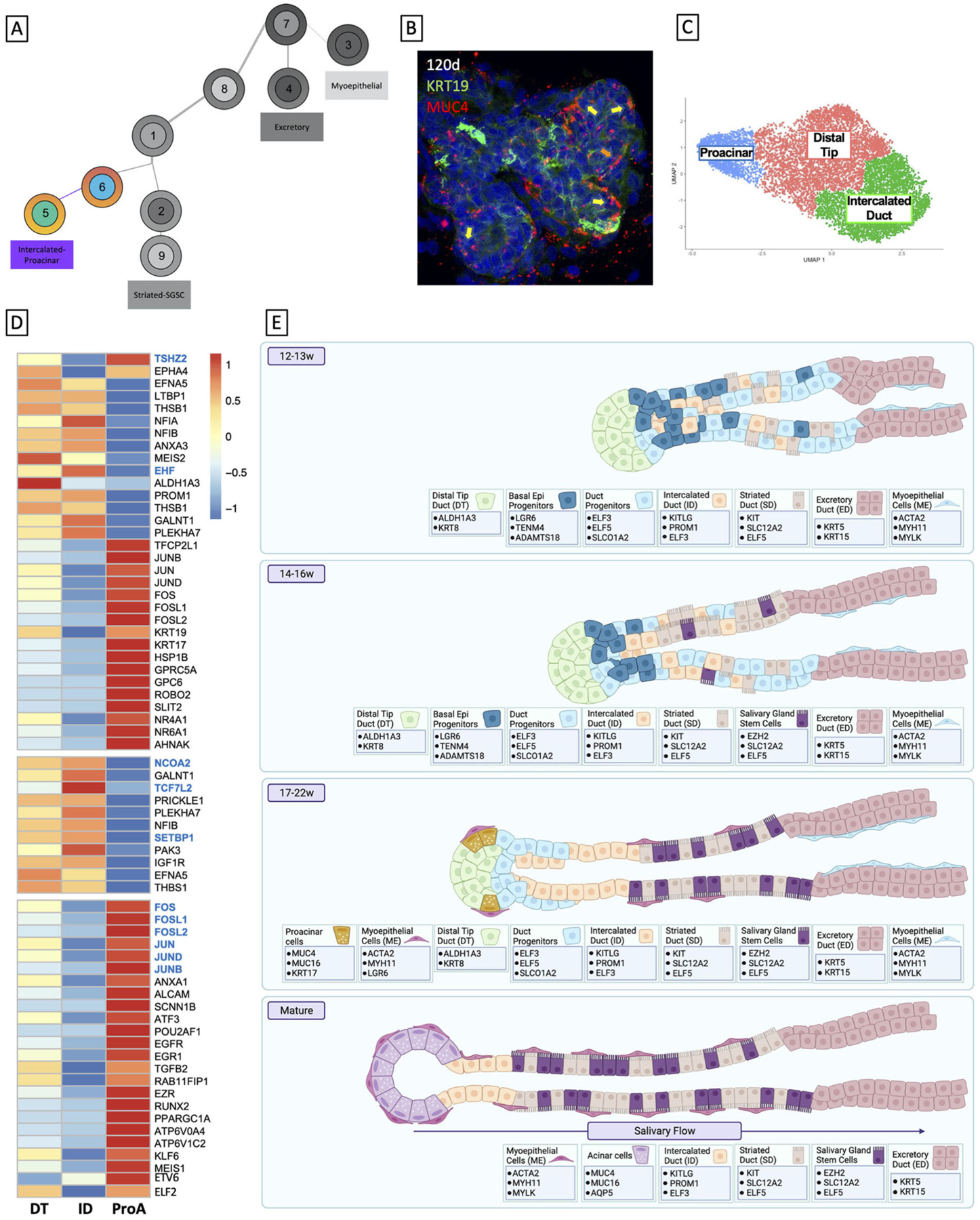
Bioinformatic analysis introduces new potential regulators of acinar differentiation. **(A)** Trajectory analysis links ID (cluster 6) and a combined distal tip-proacinar cluster (cluster 5). **(B)** MUC4, acinar-associated mucin, was observed in tissues after 19w. **(C)** The plot of subset ID, distal tip, and proacinar cells. **(D)** Transcriptional analysis shows that DT shares significant transcriptional overlap with ID, and some limited transcriptional overlap with proacinar cells. CHEA3 analysis based on top genes in each cluster shows that TSHZ2 and EHF are responsible for the transcription of many tops expressed factors in the distal tip, while several AP-1 transcription factors drive top expression in the distal tip. **(E)** Our data have identified the presence of various progenitors and previously uncharacterized cell populations at different stages of development. Created using BioRender.

A longstanding question in salivary gland development is what drives acinar differentiation. The tissues within our dataset are not yet mature enough to have mature acinar cells, therefore AMY1A and related salivary amylases, and other mature acinar markers like MIST1 are not yet expressed. Previous studies in mouse models have demonstrated that at some point in development, the distal tip of the duct reorganizes to produce acini (27). In humans, this process occurs comparatively slowly; salivary glands continue to develop through 28 weeks, at which time the acini begin to secrete products (136). However, maturation continues through postnatal stages (137), making studying their developmental trajectory difficult. Interestingly, tip reorganization has been observed as early as 16 weeks (136), suggesting that, although we do not likely have acinar cells to evaluate, we are within the appropriate timeframe to evaluate the transition from distal tip duct to acinar cells. In our dataset, we observed a small population of cells that clustered along with the distal tip that expressed markers that have previously been observed in developing acinar tissues (MUC1, MUC4, MUC16) that appears in the 17–19w timepoint (Figure 1E, cluster 5, Figure 5A). Interestingly, though we do not have an expression of mature acinar markers, the proacinar cells (tip of cluster 5) also uniquely express the related factor BHLHE40 (Supplementary Figure 5D). MIST1, also called BHLHA15 has a predicted binding site for BHLHE40 in its promoter region, suggesting that expression of BHLHA15 could be imminent. Moreover, other genes that have been classically associated with mature acini exhibit non-acinar expression patterns in the developing human glands. MUC5B is broadly expressed in ductal clusters (1,2,5,6,9), while MUC7 expression is more restricted, occupying clusters 1, 2, and 6 (DP, SD, and ID, respectively). AQP5 expression is low but is primarily restricted to the striated duct (cluster 2), a phenomenon that has previously been observed in developing mouse tissues (138, 139).

Consistent with our observation of MUC4 in proacinar cells, we observed limited MUC4+ cells in tissues 19 weeks and older (Figure 5B). To separate the distal tip and proacinar cell types, we subset cluster 5 and, predictably, observed two clusters (Supplementary Figure 5A). Pseudotime analysis suggests that 5a, the distal tip, bifurcates back into itself and into cluster 5b, the proacinar cells (Supplementary Figure 5B). Gene module analysis revealed 4 gene modules (Supplementary Figure 5C), though only modules 1 and 2 harbored all the highly expressed genes in the clusters, with module 1 occupying cluster 5b and module 2 occupying 5a, suggesting that there were no further subsets between these groups. Because ID shares significant gene expression with the distal tip duct, we evaluated ID along with distal tip and proacinar (Figure 5C) to better highlight genes within the distal tip group that might be more critical for reorganization toward proacinar groups. KEGG analysis of the proacinar group (Supplementary Figure 5E) produced salivary secretion as the top function of that group, while gene ontology analysis (Supplementary Figure 5F) ranked terms including glandular development, fluid homeostasis, and various metabolic responses among the top terms associated with the proacinar group. The gene ontology of the distal tip (Supplementary Figure 5G) was much more general, with top-ranked terms including growth, and anatomical structure morphogenesis. Differential expression analysis and ChEA3 transcription factor prediction analysis (Figure 5D) confirmed that the distal tip duct and ID exhibit significant transcriptional overlap, except for two genes that are much more highly expressed in the distal tip duct compared to ID. The first one, MEIS2, has been shown to be highly expressed in salivary glands (140) and previous studies have shown that Meis2^−/−^ mice exhibit severe craniofacial malformations, including absent or underdeveloped salivary glands in at least 33% of the animals surveyed (141). The other, ALDH1A3, is a NAD-dependent aldehyde dehydrogenase that catalyzes the formation of retinoic acid (142). Previous studies have found that expression of retinoic acid-responsive genes arises earlier than most salivary gland-specific genes and persists throughout development (143), consistent with the ongoing presence of the distal tip as the gland continues to develop.

The ChEA3 analysis revealed that two transcription factors were likely responsible for the bulk of the high gene expression in the distal tip. The first, TSHZ2, was responsible for the expression of ephrin RTK EPHA4 and its ligand EFNA5 (144), among others. Overexpressing TSHZ2 in mammary glands in mice accelerated their development while preventing malignancy (145), suggesting that this factor may help to regulate the ongoing growth of the distal tip. The other transcription predicted transcription factor was EHF, an epithelial-specific Ets-family transcription factor. This gene is not only responsible for the transcription of genes that are unique to that cluster, but also genes that are expressed both in the distal tip and then increase in the proacinar group (Supplementary Figure 5H). Most intriguing among these is the host of AP-1-related genes: JUN, FOS, etc. Among the salivary epithelium, these genes are highly enriched in the proacinar group. A recent study (146) discovered that EHF exhibits anti-cooperative DNA binding with AP-1 factors; once EHF drives the expression of AP-1 factors, the combination of FOS/JUN factors exhibits a conformation that preferentially excludes EHF from binding to downstream targets, but is compatible with others, driving a differential regulation of Ets-responsive genes. Since distal tip reorganization occurs later in development, we used Feature Scatter to evaluate how many cells in the distal tip co-expressed EHF and AP-1 factors at each age group (Supplementary Figure 5I). Consistent with our hypothesis, we observe few cells in the 12–13w distal tip that co-express EHF and FOS/JUN, despite the cells in that cluster at that age group being abundant. However, in the 17–19w timepoint, we observed an increase in these co-expressing cells (Supplementary Figure 5I), bolstering our hypothesis that AP-1 factors can exclude EHF binding to promoter regions, thereby driving a fate change toward proacinar types. Incidentally, the proacinar group exhibits a high expression of two other Ets family transcription factors: ETV6 and ELF2, suggesting that these factors may be important in specifying proacinar cells from the distal tip. As transcription factors, AP-1 components can be driven by multiple upstream pathways, but KEGG analysis of the distal tip group (Supplementary Figure 5J) suggests that in the salivary gland, this upstream driver might be ERBB signaling mediated through ERBB4 and EGFR. Previous studies have implicated Wnt signaling (33) and the transcription factor Sox2 (147) in acinar differentiation, both of which have also been shown to drive AP-1 signaling (148, 149), so future functional studies will be required to verify whether AP-1 signaling is the link.

Together, our studies have added some clarity to human fetal salivary gland development and laid a foundation for further *in vitro* developmental studies. At 12–13 weeks, salivary glands are in the early pseudoglandular stage, comprised primarily of non-lumenized ducts with progenitor identity. In the 14–16 weeks timepoint, tissues move into the late pseudoglandular phase, exhibiting more branching and lumenization, and by 17 weeks, cells have entered the canalicular stage, wherein we are able to observe distinct branching, widespread lumenization, and the defined ductal types. By 19 weeks, we begin to observe proacinar cells, and branching becomes more complex and defined through 22 weeks. We have also been able to identify genes that mark cell groups at each stage, which can be used as benchmarks for *in vitro* studies (Figure 5E).

## DISCUSSION

Here we report the findings of the first comprehensive single-cell analysis of the human fetal salivary gland. In our study, we were able to identify all ductal subtypes (excretory, striated, intercalated, distal tip), their progenitors (basal epithelial progenitors, duct progenitors), and myoepithelial cells and acinar precursors (proacinar cells), as well as a previously undescribed stem cell-like group. We were able to use this information to infer the likely developmental trajectory of these groups during development, and through differential gene expression analysis, ligand-receptor and cell-cell communication analysis, and transcription factor prediction analysis, we were able to elucidate pathways that drive each change in cell fate, as well as specific identifiers and critical transcription factors for each cluster. In the course of this study, we have been able to uncover valuable human paradigms that will be critical for the development of both salivary gland organoids as well as diagnostic tools and therapeutics for salivary gland cancers and other disorders and can provide useful information for others studying the development of other epithelial branching organs. One limitation of studying salivary gland development in human fetal tissue is that the human salivary gland at 23 weeks gestation (the latest point at which it is possible to procure human fetal tissue) is still quite undeveloped; while all structural components exist, and acinar precursors are present, functional acini will not appear for an additional 4 weeks. However, our study lays the groundwork to develop *in vitro* salivary organoid models that can be used to further study acinar development and function.

### A First-of-Its-Kind Human Comparison to Existing Mouse Developmental Trajectories

Other groups have conducted single-cell RNA sequencing on salivary glands and those studies have made headway in our understanding of cell heterogeneity during development and comparative studies between salivary glands (43–45, 150), there remain questions with regard to identifiers for specific ductal cell subtypes (i.e., intercalated and striated duct) and pathways driving fate changes for these cell types during development. In a comparable study, murine developmental timepoints E12, E14, and E16 were analyzed, which correspond in human gestation to roughly 8–10 weeks gestation, 12–14 weeks gestation, and 20–22 weeks gestation, respectively. Broadly, the trajectory presented in this study aligns with ours. We have an early population of progenitors that yields first the excretory duct and myoepithelial cells and then later gives rise to other types of SG ducts. They also observe that one of the populations of acinar cells is derived from an intercalated duct, as we see in our data. Our trajectories exhibit more similarity earlier on in the trajectory, in that they have “Krt19+ duct” which is transcriptionally similar to the BEPs identified in this study, though we observe that all ductal cell types are KRT19+. That population gives rise to what Hauser refers to as “basal duct”, denoted in our study as excretory duct, and, later on, myoepithelial cells, a phenomenon we also observe. Intriguingly, consistent with our *in vivo* findings, this study observes two populations of myoepithelial cells, though this is not directly addressed. Moreover, at later postnatal timepoints, the Krt19+ duct yields more mature duct types (striated and intercalated), similar to the bifurcation from duct progenitors observed in our study. Additionally, they only observe mature acinar cells postnatally. Considering the ongoing maturation of human salivary glands, we would also expect to see mature acini postnatally.

We also observe differences between the trajectory generated in our study and the murine study. At E16, which is more similar to 20–22w gestation in humans, they still do not observe more mature types; these appear at much later points (postnatally), while we observe different types of duct present at earlier timepoints (17–19w). Finally, we observe a small population of proacinar cells by 19w, while the mouse study found multiple populations of proacinar cells, but only in postnatal samples. Comparison between these studies provides the first insight into both conserved and divergent aspects of murine and human salivary gland developmental trajectories.

### A Fresh Look at the Definition of Stem Cells in the Salivary Gland

A proper definition and location for salivary gland stem cells, or even whether such a population indeed exists, has been a lingering question for some years, and more and more, we are coming to terms with the idea that our standard definition of tissue stem cells may not apply in the case of salivary glands (151). Most studies employing “salivary gland stem cells” have identified them functionally, by isolating a salivary gland, dissociating the tissue to a single-cell suspension, and culturing them (152–155). These cultures have then been driven to salispheres and organoids that can express acinar markers, but whether these were the result of actual salivary gland stem cells, or the result of a heterogeneous population of cells with predetermined identities remains unclear, since the resulting cultured populations were not molecularly characterized or traced back to *in vivo* tissues. Attempts to identify stem cells *in vivo* by dissecting label-retaining cells have been hampered by the fact that the salivary gland is a tissue with very slow turnover (156).

Lineage tracing revealed that homeostatic maintenance of acinar tissues occurred through self-duplication rather than replacement by a stem cell population (116), making it more likely that there is not a traditionally defined population of stem cells. This is critical because salivary glands are extremely sensitive to radiation therapy, with patients losing 50–60% of salivary flow within the first week of radiotherapy (157, 158) due to acinar ablation (159) and fibrosis (160), damage that sometimes recovers 6 months to a year after ending radiation therapy, but which can be permanent (161–163). It is known that radiation damage causes p53-mediated apoptosis (164), but that some cells can survive by employing a practice known as reversible quiescence (165–167), suggesting that recovered salivary gland function may result from salivary gland stem cells capable of entering reversible quiescence.

More recently studies have shifted to looking for stem cells within the ductal tissue. One study found that although duct and acinar homeostasis is maintained separately, following radiation damage, both duct and acinar cells can produce acinar cells (168). Another study observed that more diverse populations of progenitors were responsible for replacing different parts of an irradiated gland, with Krt14+/Kit− cells yielding duct and Krt14+/Kit+ cells yielding acinar cells (78). However, this study didn’t make it clear whether isolating those Krt14+/Kit+ cells could behave like stem cells *ex vivo*. Other studies have suggested roles for Sox2-mediated acinar regeneration (169), but this repair is suggested to be mediated through nerve interaction, however, nerves also experience genotoxic shock during radiation therapy (170), leaving the more stable ducts as the most likely to harbor reparative stem cell populations.

### New Insights Into Important Fate Drivers in Human Salivary Glands

Gain- and Loss-of-function studies in murine salivary glands over the past 20 years have identified important signaling pathways and some cell-type-specific identifiers, but our study represents the first time that these findings can be compared to human development. We observed consistency with regard to the importance of FGF and WNT signaling and laminin-integrin interactions. FGF10 and FGF7 were heavily and uniquely expressed by the mesenchyme and its primary receptor FGFR2 was broadly expressed throughout the salivary epithelium, consistent with mouse studies that found that disruption of Fgf10 resulted in the salivary gland aplasia (29, 171). Moreover, we observe specific enrichment of FGFR1 in the BEPs, myoepithelial cells, and the excretory duct, the earliest clusters to develop, consistent with previous studies that showed it as critical for branching morphogenesis (172). We also observed widespread expression of noncanonical WNT ligand WNT5B and WNT target TFCP2L1, which have also previously been shown to be necessary for branching morphogenesis (32) and which were also found to drive Ectodysplasin mediated lumenization (173–175).

A novel finding for us was the potential importance of TGFβ in salivary gland development. Some limited studies have observed disruption to salivary gland development in Tgfb1 knockout mice (176), but the bulk of studies in salivary gland point to a role for TGFβ in driving fibrosis in response to injury (177, 178). Recent data (79) suggests that TGFβ plays a role in tube formation and differentiation in mammary, intestinal, liver, kidney, and lung tissues, which share developmental aspects with salivary glands. Our studies suggest TGFβ activity is involved in duct progenitor to straited duct lineage. One potential role for TGFβ in driving striated cell identity would be to set up the critical calcium sensing in the SD (179).

Studies of transcription factor Ascl3 (180) revealed that it marked a duct-specific population of cells that was able to regenerate both duct and acinar tissues. While we do not observe expression of ASCL3 in our human dataset, ChEA3 transcription factor prediction returned ASCL3 as a highly ranked predicted transcription factor in both SD and ID based on their highly expressed genes, suggesting that either ASCL3 expression was too low to detect or there may be a human-specific Ascl3− like transcription factor that has yet to be identified. Moreover, Ascl3 studies in adult mice found that all Ascl3+ duct cells also expressed Slc12a2, while Ascl3− cells did not (115). Moreover, Ascl3+/Slc12a2+ exhibited both ductal and acinar plasticity, further bolstering the notion that the “stem cells” of the salivary gland are localized to the SD.

Finally, though our single-cell seq dataset stops at 19w gestation, we were able to validate some of our findings in an older tissue, ~22w wherein we observed an interesting phenomenon in the ED. As we previously discussed, basal duct marker KRT15 is highly enriched in our dataset. Using this marker in the 22w tissue, we observed that protrusions from the basal epithelium reached through to the luminal epithelium (Figure 2M, yellow arrows). This phenomenon has been previously described in the epidydimal epithelium; wherein the basolateral epithelium sent forth long epithelial projections toward the cells on the luminal side of the duct that cross tight junctions (181). They further found that these projections served as luminal hormone sensors that sensed the luminal levels of Angiotensin II to check for luminal fluid balance. While this specific phenomenon has not been described in other tissues, mammary glands are known to have luminal hormone sensors (182). This finding suggests that there may be yet undescribed functions for the excretory duct that may be important either in gland maturation or function and merits further investigation.

### Impact on Organoid Development and Cancer Research

Our studies lay the groundwork to further our understanding of not only salivary gland development but also salivary gland pathologies and organoid development. To date, most salivary gland organoid studies rely on the use of cells isolated from the developed salivary glands (154, 183–185). While these can be useful in a variety of research and pharmaceutical applications, they can also present some significant shortcomings. Therefore, the ideal course of action is to develop an organoid from induced pluripotent stem cells that can have both research and therapeutic potential. The results reported in this study can facilitate the development of such an organoid.

iPSC-derived organoids can have major implications for an ongoing study of development. While many gain- and loss-of-function have been conducted in mice, this is impossible in humans. However, with an iPSC-derived organoid protocol, we would be capable of conducting the same types of functional studies with precision control over when a gene is overexpressed or ablated. This organoid system could allow us to take these studies beyond basic transcriptional studies, enabling the creation of salivary gland disease models and facilitating the investigation of epigenetic regulation and non-coding RNAs in development, function, and degeneration. It can also facilitate the study of cutting edge tools and therapeutics such as hyper-specific AI designed proteins (186, 187). This can be exceedingly useful to better understand the autoimmune salivary gland degenerative disease known as Sjögren’s Syndrome, the cause of which has long evaded elucidation, but which has recently been shown to have significant epigenetic modification compared to healthy individuals (188).

The discoveries reported here can also have implications for cancer diagnosis and treatment. To date, standard treatments for salivary gland cancers include surgical resection, radiation therapy, and chemotherapeutics (189). As with all cancers, resection may allow for the recurrence of cancer, therefore necessitating radiation and/or chemotherapy in order to be effective. Moreover, surgical recommendations state that facial nerves may also need to be resected, which can lead to significant complications later on with salivation from remaining glands as well as facial movement. As previously discussed, the necessity of radiation therapy can ablate acinar cells in unaffected glands. Chemotherapeutics for salivary glands remain a poor option; the last clinical trial looking into doxorubicin to treat advanced carcinoma was conducted in 1996 and reported a meager 27% response rate (190). More recent studies looking at antibody therapies have been equally disappointing; although anti-EGFR and anti-HER2 therapies make some improvement, many patients ultimately develop resistance and do not reach remission (191, 192). Though other therapeutics are under investigation, a better understanding of critical genes that can drive a terminally differentiated salivary gland duct or acinar cell back to a stem-like state would likely facilitate the development of more effective therapeutics (193).

A better understanding of salivary gland subtype markers can also facilitate diagnosis. Recently there have been several instances of misdiagnosing a primary salivary duct carcinoma as primary breast cancer. One specific case report described a patient who had previously been diagnosed and treated for salivary duct carcinoma and later presented with a mass in the left anterior chest (194). Radiological analysis suggested a novel diagnosis of primary breast cancer because metastasis of salivary tumors to the breast had never been previously reported and the histological analysis had the appearance of breast tissue. Moreover, high-grade salivary and breast acinic carcinomas, because they share developmental paradigms and various tissue markers, are said to be extremely similar in their diseased state. In the absence of better markers, the salivary carcinomas are diagnosed as salivary over breast simply because they “lack all the cardinal molecular features of conventional triple-negative breast cancer” and are “underpinned by hotspot mutations”, effectively, mutations that are sometimes seen in salivary carcinomas (195), but many of which are not characterized as to their role in salivary gland function or development (196). However, this method of diagnosis has repeatedly proven to be faulty; in addition to the case report above where a salivary cancer was diagnosed as primary breast cancer, there have been reports in the opposite direction, where a primary acini breast carcinoma lacking those cardinal breast cancer features was diagnosed and treated as a primary salivary acinic carcinoma, unfortunately resulting in the death of the patient (48). These reports make it increasingly obvious that there is a need for better characterization of developing salivary glands to facilitate the appropriate diagnosis of salivary vs. breast carcinomas and to develop effective targeted chemotherapeutic treatments.

## CONCLUSION

In conclusion, we have conducted a single-cell analysis on developing salivary glands in humans. In doing so, we were able to augment what is known about the developing salivary gland, what signaling pathways are involved in cell fate bifurcations of ductal cell types, and what genetic markers identify different populations. We also identified a novel stem cell-like population derived from the SD that may represent stem cells that have the capability of regenerating acinar groups. We also uncovered non-traditional roles for myoepithelial cells during development that diverge from their preconceived function of compressing acinar cells to expel saliva. Our findings can be used to facilitate organoid development for therapeutic use or disease modeling.

## Supplementary Material

Data_Sheet_1_Sci-Seq of Human Fetal Salivary Tissue Introduces Human Transcriptional Paradigms and a Novel Cell Population

Image_2_Sci-Seq of Human Fetal Salivary Tissue Introduces Human Transcriptional Paradigms and a Novel Cell Population **Supplementary Figure S2** | **(A-C)** Density plotted by age of BEP subset. **(D)** A Heatmap of the top 300 genes per age group in myoepithelial cells demonstrates a transcriptional shift from early immature myoepithelial types to later mature myoepithelial types. **(E)** Venn Diagram of transcriptional overlap between different age groups of myoepithelial cells. **(F)** Log expression of LGR6 in the salivary epithelium exhibits expression in BEPs (purple arrows) and myoepithelial cells (pink arrow). **(G)** Log expression of LGR6 in BEP subset. **(H)** Top expressed genes in BEPs and DPs are both also expressed in Epithelial Progenitors. **(I)** Gene ontology analysis of excretory duct.

Image_1_Sci-Seq of Human Fetal Salivary Tissue Introduces Human Transcriptional Paradigms and a Novel Cell PopulationImage_2_Sci-Seq of Human Fetal Salivary Tissue Introduces Human Transcriptional Paradigms and a Novel Cell Population **Supplementary Figure S1** | Cluster identification. **(A)** A Heatmap of the top 500 genes per cluster mimics the predicted developmental trajectory, showing that early groups (Basal Epithelial Progenitors, Excretory Duct, Myoepithelial Cells) exhibit more transcriptional overlap than later duct types. **(B)** Top gene analysis shows highly expressed genes in each cluster of the salivary epithelium and the support tissues **(C)** from 12–19 weeks.

Image_5_Sci-Seq of Human Fetal Salivary Tissue Introduces Human Transcriptional Paradigms and a Novel Cell Population **Supplementary Figure S5** | **(A)** Subset of cluster 5 yielded two clusters. **(B)** Pseudotime analysis suggests that cells in the distal tip give rise to proacinar cells and to themselves. **(C)** Module analysis showed two significant gene modules for the cluster 5 subsets. **(D)** Log expression distribution of mature acinar markers **(E)** KEGG analysis for the proacinar groups shows salivary secretion as the highest scored category. **(F)** Gene ontology analysis for the proacinar group. **(G)** Gene ontology analysis for the distal tip duct. **(H)** Expression of transcription factor EHF and its transcriptional targets shows that several are expressed exclusively in the proacinar group while others are expressed in both the distal tip and proacinar group. **(I)** Feature scatter shows the number of cells in the distal tip at 12–13w or 17–19w timepoints that co-express either EHF and FOS, EHF and JUN, or FOS and JUN. The abundance of these co-expressing cells increases at the 17–19 weeks timepoint when distal tip reorganization toward proacinar cells is occurring. **(J)** The plot of KEGG analysis for ERBB signaling suggests that AP-1 signaling in the reorganizing distal tip may be mediated through ERBB signaling.

Image_3_Sci-Seq of Human Fetal Salivary Tissue Introduces Human Transcriptional Paradigms and a Novel Cell Population **Supplementary Figure S3** | **(A)** Isolated plot of striated (SD), intercalated (ID), and duct progenitors (DP). **(B-D)** Log expression of top genes in striated **(B)** and intercalated **(C,D)** ducts. **(E)** ChEA3 predicted the top 50 transcription factors based on top gene expression. **(F)** Gene ontology analysis of SD. **(G)** Gene ontology analysis of ID. **(H)** The plot of enriched factors related to active TGFβ pathway.

Image_4_Sci-Seq of Human Fetal Salivary Tissue Introduces Human Transcriptional Paradigms and a Novel Cell Population **Supplementary Figure S4** | **(A-C)** Density plotted by age in SD subset. **(D)** The plot of isolated SD (cluster 2) and Salivary gland stem cells (SGSC) (cluster 9). **(E)** The plot of expression scores for each identified gene module. **(F-H)** The SGSC cluster appears among the latest tissue types according to pseudotime **(F)** and exhibits a high proliferation index compared to other clusters **(G)**. It also exhibits an enriched expression of EZH2 **(H)**. **(I)** Gene ontology analysis of SGSC cluster.

## Figures and Tables

**TABLE 1 |:** Antibodies and probes used.

Antibody/Probe	Gene name	Species	Dilution	Company (Catalog #)
Antibodies				
Krt19	KRT19	Preconjugated AF488	1:100	R&D Systems (IC3506G)
Phalloidin	N/A	Preconjugated AF 568	1:400	Thermo Fisher Scientific (A12380)
Acta2	ACTA2	Preconjugated AF 647	1:50	R&D Systems (IC1420R-100UG))
Krt15	KRT15	Rabbit	1:1000	Sigma (HPA023910–25UL)
Atpb	ATPB	Mouse	1:100	Abcam (ab14730)
Nkccl	SLC12A2	Rabbit	1:100	Cell Signaling Technologies (8351S)
Ezh2	EZH2	Rabbit	1:100	Cell Signaling Technologies (5246S)
Muc4	MUC4	Mouse	1:100	Thermo Fisher Scientific (35–4900)
RNA scope probes				
LGR6	LGR6	n/a	1:50	ACDBio (410461-T5)
SLC12A2	SLC12A2	n/a	1:50	ACDBio (564681-T3)
MMP16	MMP16	n/a	1:50	ACDBio (473931-T1)
THSD4	THSD4	n/a	1:50	ACDBio (892041-T2)

## Data Availability

The data presented in the study are deposited in the GEO Database repository, accession number GSE184749.
