# Supplementary figures and images for: Sci-Seq of Human Fetal Salivary Tissue Introduces Human Transcriptional Paradigms and a Novel Cell Population
Source: Front Dent Med. Author manuscript; Available in PMC 2022 Dec 19. (PMC9762771; doi:10.3389/fdmed.2022.887057)

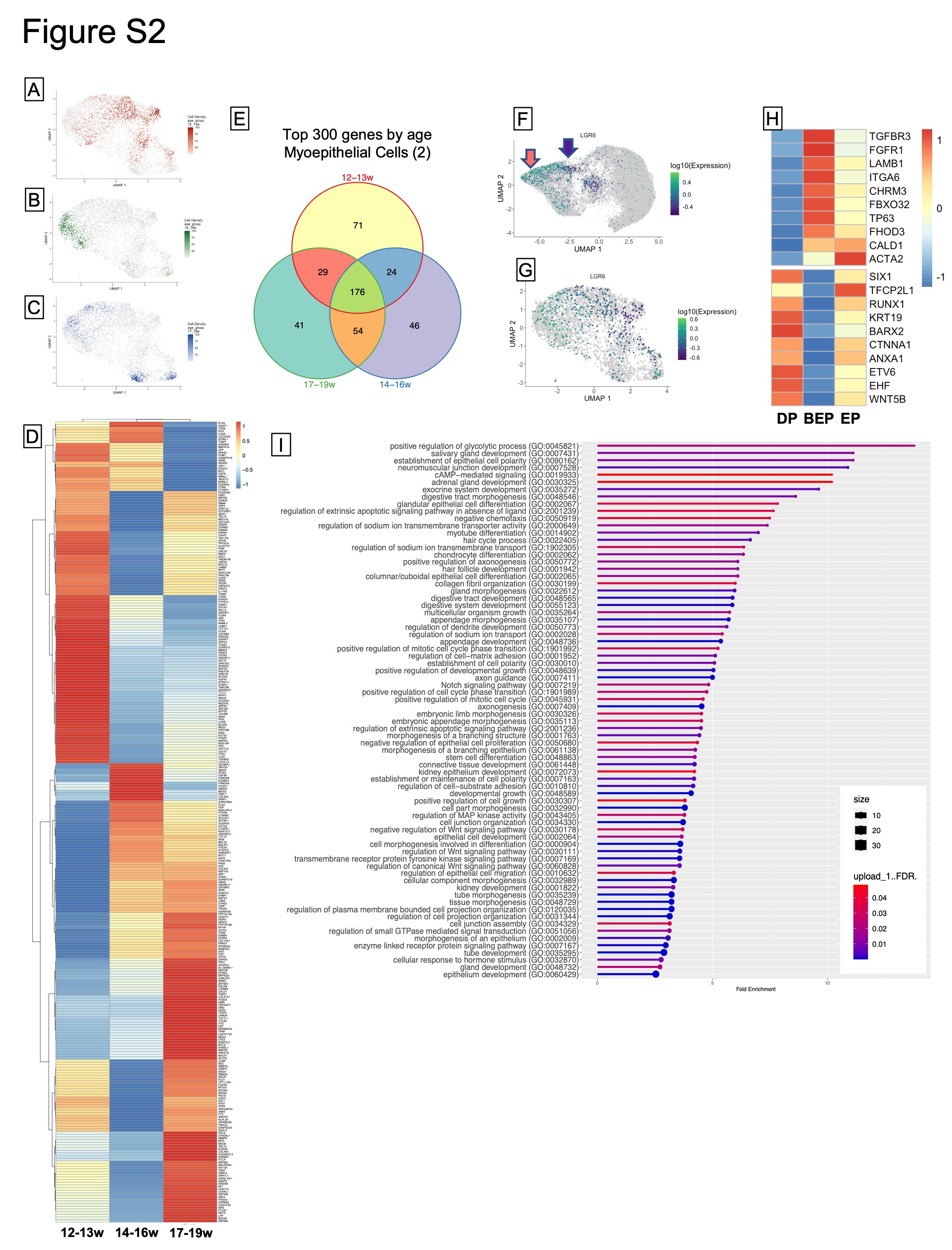

Supplement: Image_2_Sci-Seq of Human Fetal Salivary Tissue Introduces Human Transcriptional Paradigms and a Novel Cell Population — Supplementary Figure S2 | (A-C) Density plotted by age of BEP subset. (D) A Heatmap of the top 300 genes per age group in myoepithelial cells demonstrates a transcriptional shift from early immature myoepithelial types to later mature myoepithelial types. (E) Venn Diagram of transcriptional overlap between different age groups of myoepithelial cells. (F) Log expression of LGR6 in the salivary epithelium exhibits expression in BEPs (purple arrows) and myoepithelial cells (pink arrow). (G) Log expression of LGR6 in BEP subset. (H) Top expressed genes in BEPs and DPs are both also expressed in Epithelial Progenitors. (I) Gene ontology analysis of excretory duct. [file NIHMS1855378-supplement-Image_2_Sci-Seq_of_Human_Fetal_Salivary_Tissue_Introduces_Human_Transcriptional_Paradigms_and_a_Novel_Cell_Population.jpeg]

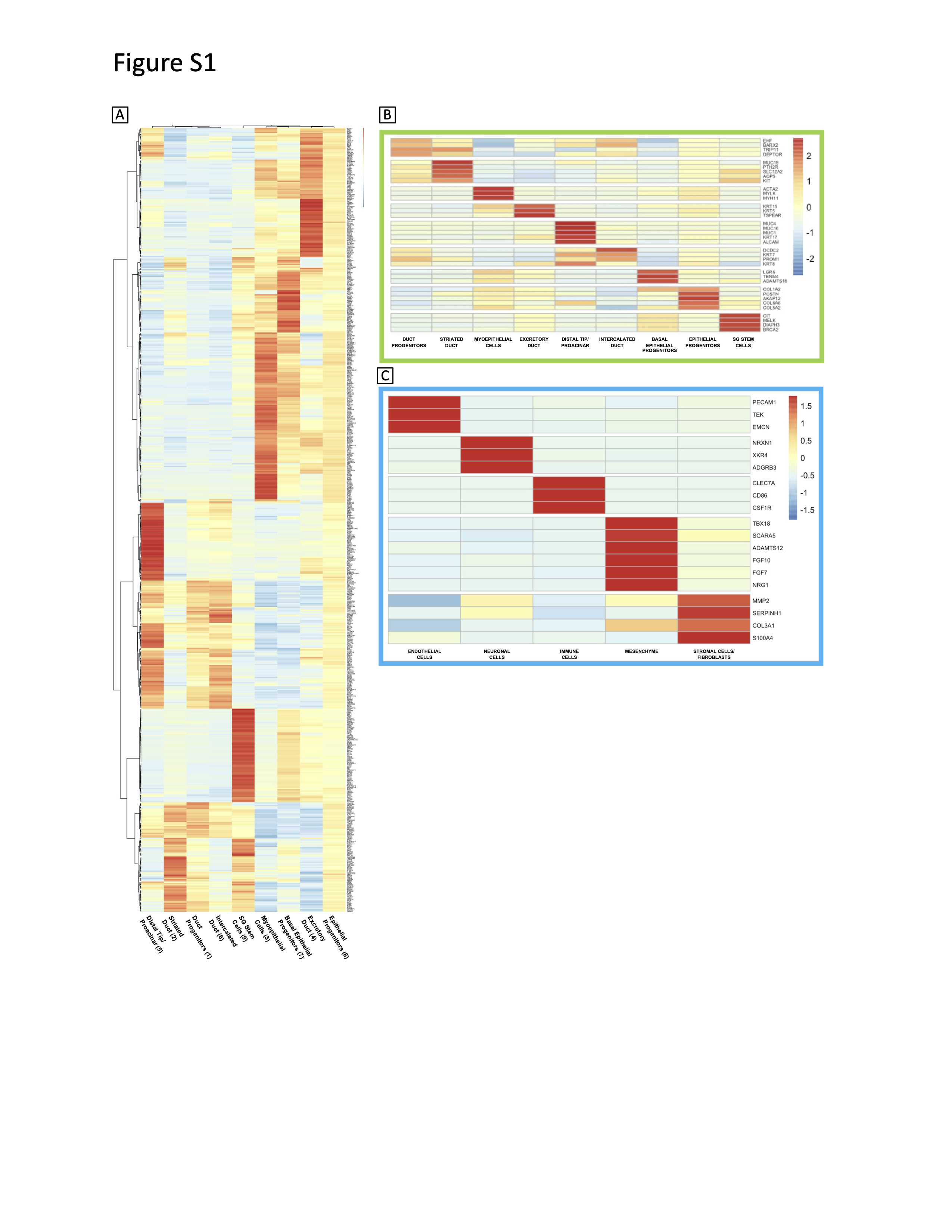

Supplement: Image_1_Sci-Seq of Human Fetal Salivary Tissue Introduces Human Transcriptional Paradigms and a Novel Cell PopulationImage_2_Sci-Seq of Human Fetal Salivary Tissue Introduces Human Transcriptional Paradigms and a Novel Cell Population — Supplementary Figure S1 | Cluster identification. (A) A Heatmap of the top 500 genes per cluster mimics the predicted developmental trajectory, showing that early groups (Basal Epithelial Progenitors, Excretory Duct, Myoepithelial Cells) exhibit more transcriptional overlap than later duct types. (B) Top gene analysis shows highly expressed genes in each cluster of the salivary epithelium and the support tissues (C) from 12–19 weeks. [file NIHMS1855378-supplement-Image_1_Sci-Seq_of_Human_Fetal_Salivary_Tissue_Introduces_Human_Transcriptional_Paradigms_and_a_Novel_Cell_PopulationImage_2_Sci-Seq_of_Human_Fetal_Salivary_Tissue_Introduces_Human_Transcriptional_Paradigms_and_a_Novel.jpeg]

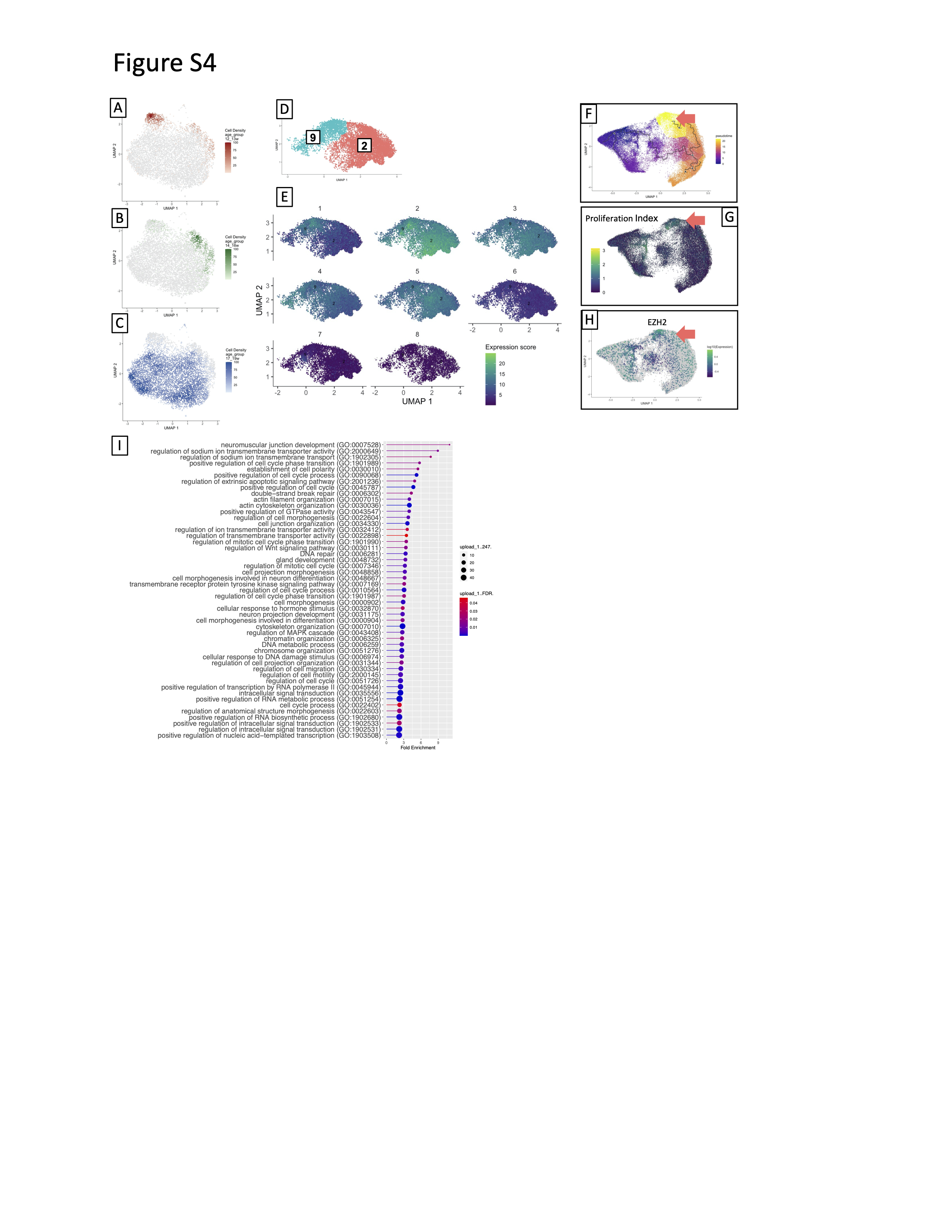

Supplement: Image_5_Sci-Seq of Human Fetal Salivary Tissue Introduces Human Transcriptional Paradigms and a Novel Cell Population — Supplementary Figure S5 | (A) Subset of cluster 5 yielded two clusters. (B) Pseudotime analysis suggests that cells in the distal tip give rise to proacinar cells and to themselves. (C) Module analysis showed two significant gene modules for the cluster 5 subsets. (D) Log expression distribution of mature acinar markers (E) KEGG analysis for the proacinar groups shows salivary secretion as the highest scored category. (F) Gene ontology analysis for the proacinar group. (G) Gene ontology analysis for the distal tip duct. (H) Expression of transcription factor EHF and its transcriptional targets shows that several are expressed exclusively in the proacinar group while others are expressed in both the distal tip and proacinar group. (I) Feature scatter shows the number of cells in the distal tip at 12–13w or 17–19w timepoints that co-express either EHF and FOS, EHF and JUN, or FOS and JUN. The abundance of these co-expressing cells increases at the 17–19 weeks timepoint when distal tip reorganization toward proacinar cells is occurring. (J) The plot of KEGG analysis for ERBB signaling suggests that AP-1 signaling in the reorganizing distal tip may be mediated through ERBB signaling. [file NIHMS1855378-supplement-Image_5_Sci-Seq_of_Human_Fetal_Salivary_Tissue_Introduces_Human_Transcriptional_Paradigms_and_a_Novel_Cell_Population.jpeg]

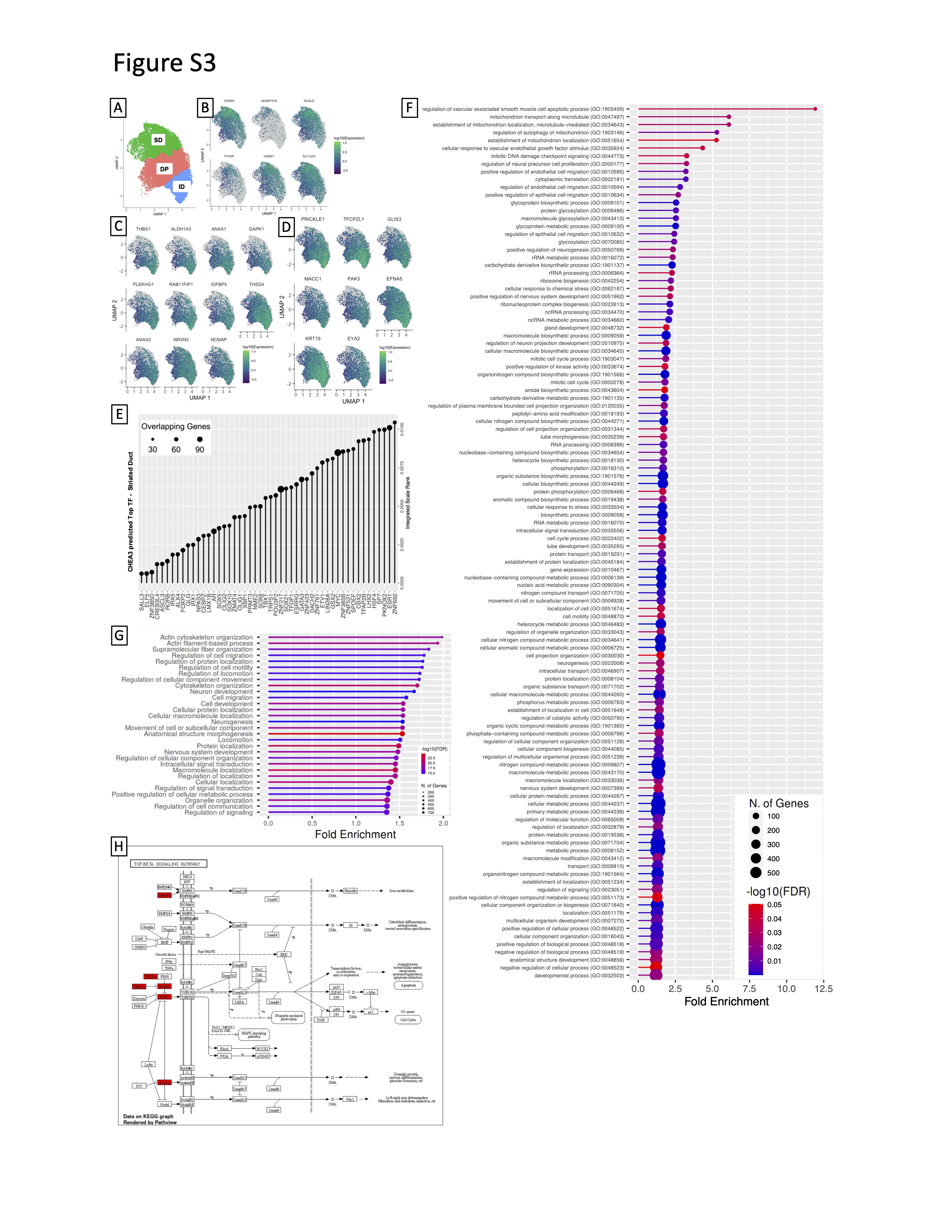

Supplement: Image_3_Sci-Seq of Human Fetal Salivary Tissue Introduces Human Transcriptional Paradigms and a Novel Cell Population — Supplementary Figure S3 | (A) Isolated plot of striated (SD), intercalated (ID), and duct progenitors (DP). (B-D) Log expression of top genes in striated (B) and intercalated (C,D) ducts. (E) ChEA3 predicted the top 50 transcription factors based on top gene expression. (F) Gene ontology analysis of SD. (G) Gene ontology analysis of ID. (H) The plot of enriched factors related to active TGFβ pathway. [file NIHMS1855378-supplement-Image_3_Sci-Seq_of_Human_Fetal_Salivary_Tissue_Introduces_Human_Transcriptional_Paradigms_and_a_Novel_Cell_Population.jpeg]

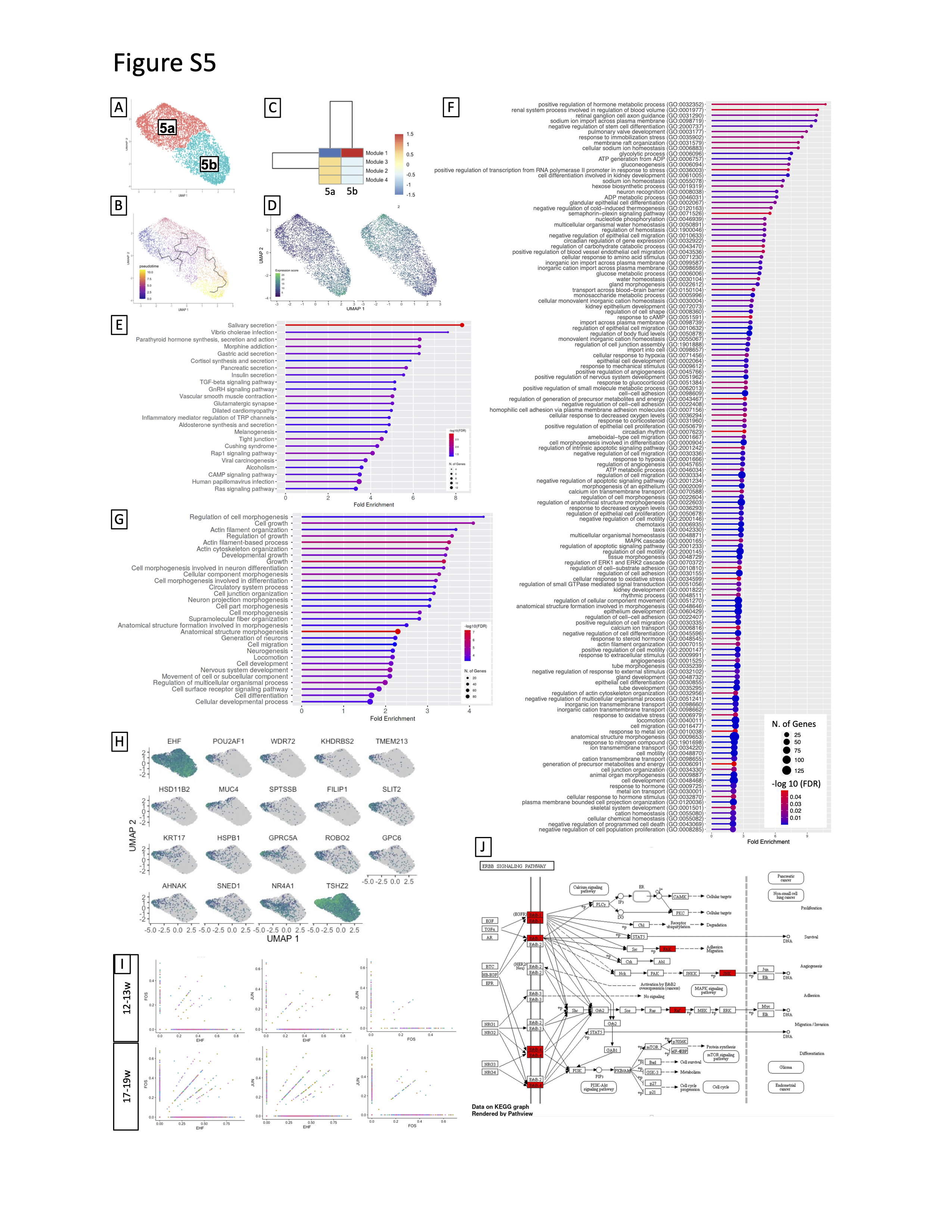

Supplement: Image_4_Sci-Seq of Human Fetal Salivary Tissue Introduces Human Transcriptional Paradigms and a Novel Cell Population — Supplementary Figure S4 | (A-C) Density plotted by age in SD subset. (D) The plot of isolated SD (cluster 2) and Salivary gland stem cells (SGSC) (cluster 9). (E) The plot of expression scores for each identified gene module. (F-H) The SGSC cluster appears among the latest tissue types according to pseudotime (F) and exhibits a high proliferation index compared to other clusters (G). It also exhibits an enriched expression of EZH2 (H). (I) Gene ontology analysis of SGSC cluster. [file NIHMS1855378-supplement-Image_4_Sci-Seq_of_Human_Fetal_Salivary_Tissue_Introduces_Human_Transcriptional_Paradigms_and_a_Novel_Cell_Population.jpeg]
